# Context-Dependent Roles of Claudins in Tumorigenesis

**DOI:** 10.3389/fonc.2021.676781

**Published:** 2021-07-20

**Authors:** Jian Li

**Affiliations:** Department of General Surgery, The Third Hospital of Mianyang, Sichuan Mental Health Center, Mianyang, China

**Keywords:** claudin, tight junction, cancer, tumorigenesis, metastasis, tumor heterogeneity

## Abstract

The barrier and fence functions of the claudin protein family are fundamental to tissue integrity and human health. Increasing evidence has linked claudins to signal transduction and tumorigenesis. The expression of claudins is frequently dysregulated in the context of neoplastic transformation. Studies have uncovered that claudins engage in nearly all aspects of tumor biology and steps of tumor development, suggesting their promise as targets for treatment or biomarkers for diagnosis and prognosis. However, claudins can be either tumor promoters or tumor suppressors depending on the context, which emphasizes the importance of taking various factors, including organ type, environmental context and genetic confounders, into account when studying the biological functions and targeting of claudins in cancer. This review discusses the complicated roles and intrinsic and extrinsic determinants of the context-specific effects of claudins in cancer.

## Introduction

The paracellular space between neighboring cells in the epithelial sheet was found to be sealed by several types of cell-cell junctions, one of which is tight junctions (TJs) (1). In 1998, claudins were identified as the major integral membrane proteins essential for TJ assembly (2, 3). Our contemporary understanding of claudins is based on their canonical barrier and fence functions. This view has expanded considerably over decades of research, following increasing evidence suggesting that claudins may be involved in signal transduction and may be causally important in tumorigenesis. Currently, the aberrant claudins in cancer are mainly believed to play a role in the disruption of the epithelial barrier and relevant signal transduction functions, and the disruption of the two functions affects nearly all aspects of tumor biology, including inflammation, growth, survival, proliferation, epithelial-mesenchymal transition (EMT), metastasis, therapy resistance and cancer stem cell (CSC) renewal. However, the contribution of the claudins to tumorigenesis, both within the same cancer type and in different cancers, varies.

In this review, I describe various studies of malignancy in cancer cells, mouse models and human tissues, and to summarize the roles claudins play in carcinogenesis, focusing on dysregulation of claudin signal transduction. Ultimately, I detail the functional complexity of claudins and summarize the intrinsic and extrinsic determinants of their context-specific effects in cancer.

## Overview of Claudins and Its Physiological Functions

Epithelial and endothelial cells in most living systems form a sheet-like structural interface between the external environment and internal compartments (4, 5). TJs are the chief intercellular junctions that act as permeability barriers and confer polarity to epithelial cells by demarcating the membrane upper and lower regions; this demarcation is accomplished by the close proximity of adjacent plasma membranes containing TJ strands formed by TJ proteins (4). As key proteins among them, claudins are a family of integral membrane proteins that make up TJ strands and act as both pores and barriers, playing pivotal roles in regulating paracellular permeability and signaling pathways (6, 7). Twenty-seven homologous claudin genes have been identified in mammals, and many more claudin proteins are selectively expressed by alternative splicing in various tissues. The structure of claudins includes four transmembrane domains (TM1-4), the intracellular N and C termini, and two extracellular loops (ECL1 and ECL2) (8, 9). Many excellent reviews have summarized the physiological functions of claudins (10, 11). Together with other proteins, claudins are assembled into TJs and play a critical role in the proper barrier function of epithelia (12). Additionally, claudins directly regulate signaling pathways or indirectly form complexes with other proteins to influence cell growth, survival, proliferation, and differentiation (10, 13, 14).

## The Roles of Claudins in Tumorigenesis

In line with their aforementioned important functions, dysregulation of claudin-mediated barrier function and signaling pathways is a recurrent event that has been demonstrated in many cancers (15–17). There are also many studies on how claudins regulate or be regulated by molecular pathways linked to cancer biology (**Supplementary Tables S1**, **S2**). Of them, claudin-1, -3, -4, -6, -7, and -18 are the most extensively studied claudins in tumors. These studies revealed a range of outcomes that reflect the complexity of claudins in terms of expression, spatial localization, tumor types, stage of disease and the roles of claudins in carcinogenesis. However, the data suggest that even if it does not function as a cancer driver, claudin dysregulation does assist in the initiation and progression of cancer and is involved in cell growth, proliferation, survival, differentiation, chemoresistance, migration, invasion and EMT. Before discussing the roles of claudins in carcinogenesis, an overview of the genetic alterations in cancer and other diseases is very useful for interpreting the results of the many cancer studies.

### *CLDN* Genetic Alterations

The advent of technologies for gene sequencing of human tumors has produced massive amounts of genetic data that should be able to determine whether mutations in claudin encoding genes can drive tumorigenesis and metastasis. However, no claudin gene mutations were identified as drivers in any tumor type in an analysis employing the IntOGen platform (https://www.intogen.org/search). This platform systematically analyses data from many sequencing projects aimed at identifying the compendium of mutational driver genes across tumor types, but it does not cover amplifications, deletions, genomic rearrangements or epigenetic silencing, which also contribute to tumorigenesis (18, 19). Unlike the number of studies of claudin gene expression, studies on claudin genetic alterations are limited, but data can be obtained from many large tumor sequencing initiatives, such as The Cancer Genome Atlas (TCGA). I searched cBioPortal (http://www.cbioportal.org) using TCGA PanCancer Atlas data, and the results are summarized in **Supplementary Table S3** (20). From the alteration frequencies and patterns, one can infer that any putative modulatory effects that claudins have on cancers are likely to be isoform- and cancer type-specific. Genetic alterations are rare in *CLDNs* other than *CLDN1* and *CLDN11*. The main alteration seen in the majority *CLDN* genes is amplification, while deep deletions are commonly present in *CLDN22-25*. An important degree of variability in any given *CLDN* gene has also been observed. For example, for *CLDN18*, gene amplification mainly occurs in lung squamous, cervical, oesophageal, head and neck, and ovarian cancer, whereas mutation is predominately found in uterine cancer, and gene fusion is predominately found in stomach cancer (21). However, whether these genetic alterations contribute to tumorigenesis and the exact protumor mechanism remains to be determined.

As germline mutations of the claudin gene family have been linked to some inherited syndromes and diseases, it should be possible to find any links to cancer or disease progression by searching patient records. Although animal models have demonstrated that genetic mutations in nearly all claudin family members can cause developmental abnormalities and diseases affecting many tissues and organs (**Supplementary Table S4**), only *CLDN1* (OMIM 603718), *CLDN5* (OMIM 602101), *CLDN14* (OMIM 605608), *CLDN16* (OMIM 603959), and *CLDN19* (OMIM 610036) have been found to cause human neonatal ichthyosis, sclerosing cholangitis, non-syndromic deafness, familial hypomagnesaemia and other symptoms (https://www.omim.org) (10). Although epidemiological studies to assess cancer incidence and progression in this unique cohort are lacking, no mutations appear to be associated with an increased propensity to develop cancer.

### Claudins as Tumor Suppressors

Genetic alterations in claudin genes are infrequently found in cancer (**Supplementary Table S3**), and there are many discrepancies between studies regarding the dysregulation of claudin expression in oncogenesis. However, decreased claudin expression in cancer is a notable trend because the majority of claudins are decreased or absent in cancers derived from tissues in which they normally highly expressed, suggesting that claudins have a function of tumor suppressor. In cell experiments, nearly all claudins have been found to be tumor suppressors in a specific context, and gain of their expression markedly inhibits tumor cell proliferation, EMT, invasion, migration, and colony formation (**Supplementary Table S2**). For nearly all of the known mammalian claudins, knockout (KO) mouse models have been established (**Supplementary Table S4**); some mice develop increased cell proliferation but not tumors (mice with claudin-4, claudin-11, or claudin-15 KO) (22–24), some develop tumors resulting from additional insults (mice with claudin-3 KO) (25), and some develop tumors spontaneously (mice with claudin-7, claudin-18.1, or claudin-18.2 KO) (26–28). Furthermore, numerous studies in the past decade have correlated low expression of claudins with advanced disease, metastasis and significantly poor prognosis (15).

**Figure 1:**
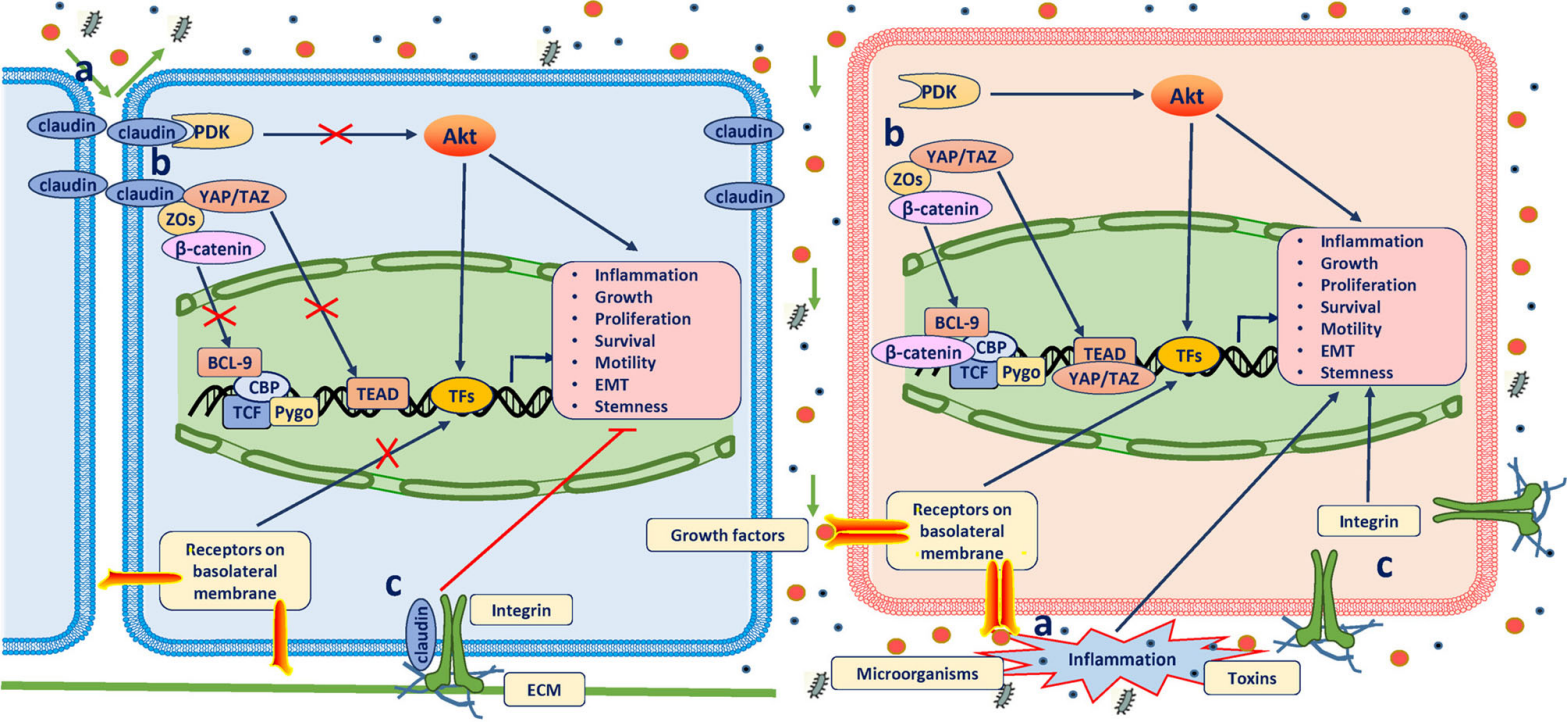
Antitumor role of claudins. The definitive mechanisms through which claudins suppress tumorigenesis have not been well established. Three possible mechanisms have been proposed: **(A)** When assembled into TJs, claudins prevent the passing of microorganisms, toxins and growth factors (GFs) through the paracellular space; otherwise, inflammation will occur, which is the most common predisposition for cancer, and GFs promote neoplastic transformation and growth by binding to their receptors on the basolateral membrane. **(B)** In TJ complexes, claudins bind and retain many other key elements of signaling pathways in the submembrane compartment to directly inhibit their activation or indirectly inhibit their activation through other scaffolding proteins (mainly ZOs). Of these molecules, YAP/TAZ, β-catenin, and PDK1 are all well-known oncogenic drivers. **(C)** Claudins in the basolateral membrane colocalize and form a protein complex with integrins to maintain epithelial cell attachment and suppress cell proliferation.

Although these studies strongly support a tumor suppressor role of claudins, the specific mechanisms underlying tumorigenesis due to claudin loss have not been well established. In view of the aforementioned functions claudins play under physiological conditions, the potential mechanisms may involve perturbations of the paracellular barrier and signal transduction (**Figure 1**). Disruption of claudin strands can allow foreign molecules and microorganisms to enter tissues through the paracellular space, causing inflammation, which is the most common predisposing factor for cancer (29). Reciprocal effects have also been described, with inflammation mediators causing dysfunction of claudins (30, 31). Moreover, disruption of claudin strands also allows growth factor infiltration into the mucosa to promote neoplastic transformation and growth (32). Therefore, most spontaneous cases of tumorigenesis result from the loss of claudins that play an important role in preventing irritant substances in specific epithelial cells (claudin-7 loss in intestinal epithelial cells and claudin 18.2 loss in gastric epithelial cells) (26, 28). Gastric acid, bacteria, and toxins can cause inflammation when they move from the apical to basal cell surfaces (33). However, deletion of some claudins results in cell proliferation lacking overt inflammation and has no effect on paracellular permeability, which suggests that non-inflammation-related pathways may contribute (34). This function likely arises from the ability of claudins in TJs to directly bind to and retain many key elements of signaling pathways in the submembrane compartment to inhibit their activation or to indirectly inhibit this activation through interactions with other scaffolding proteins [mainly zonula occludens (ZOs)]. Of these molecules, yes-associated protein/transcriptional coactivator with PDZ-binding motif (YAP/TAZ), β-catenin, and pyruvate dehydrogenase kinase 1 (PDK1) are all well-known oncogenic drivers (25, 27, 35–37). In addition, basolateral membrane claudin-7 was found to colocalize and form a protein complex with integrin β1 to maintain epithelial cell attachment and suppress cell proliferation in human lung cancer (38). Together, these studies suggest that claudins play atumor suppressor role mainly through the inhibition of inflammation and pro-oncogenic pathways. However, because many prevalent oncogenic mutations deregulate the intracellular signal transduction involving these molecules themselves and their upstream or downstream cascades, the extent to which neoplastic cells are dependent on claudin signaling is unclear.

### Claudins as Tumor Promoters

In contrast to KO mouse models, transgenic mouse models that overexpress specific claudins are limited (**Supplementary Table S4**), and only claudin-1-overexpressing transgenic mice with simultaneous adenomatous polyposis coli (APC) deletion or induced colitis have shown a role for claudin-1 in supporting tumorigenesis (39, 40). In contrast, no tumors developed in claudin-2- and claudin-6-overexpressing transgenic mice (41, 42). Therefore, evidence for the protumor role of claudins has mainly been obtained from *in vitro* studies and clinical tissues. Numerous studies have been conducted and demonstrated that overexpression of claudins plays a pivotal role in cancer biology in various cancer cell types (**Supplementary Table S2**). Additionally, the expression levels of many claudins have been found to be higher in various tumor tissues than in their normal adjacent tissues (15, 16, 43).

**Figure 2:**
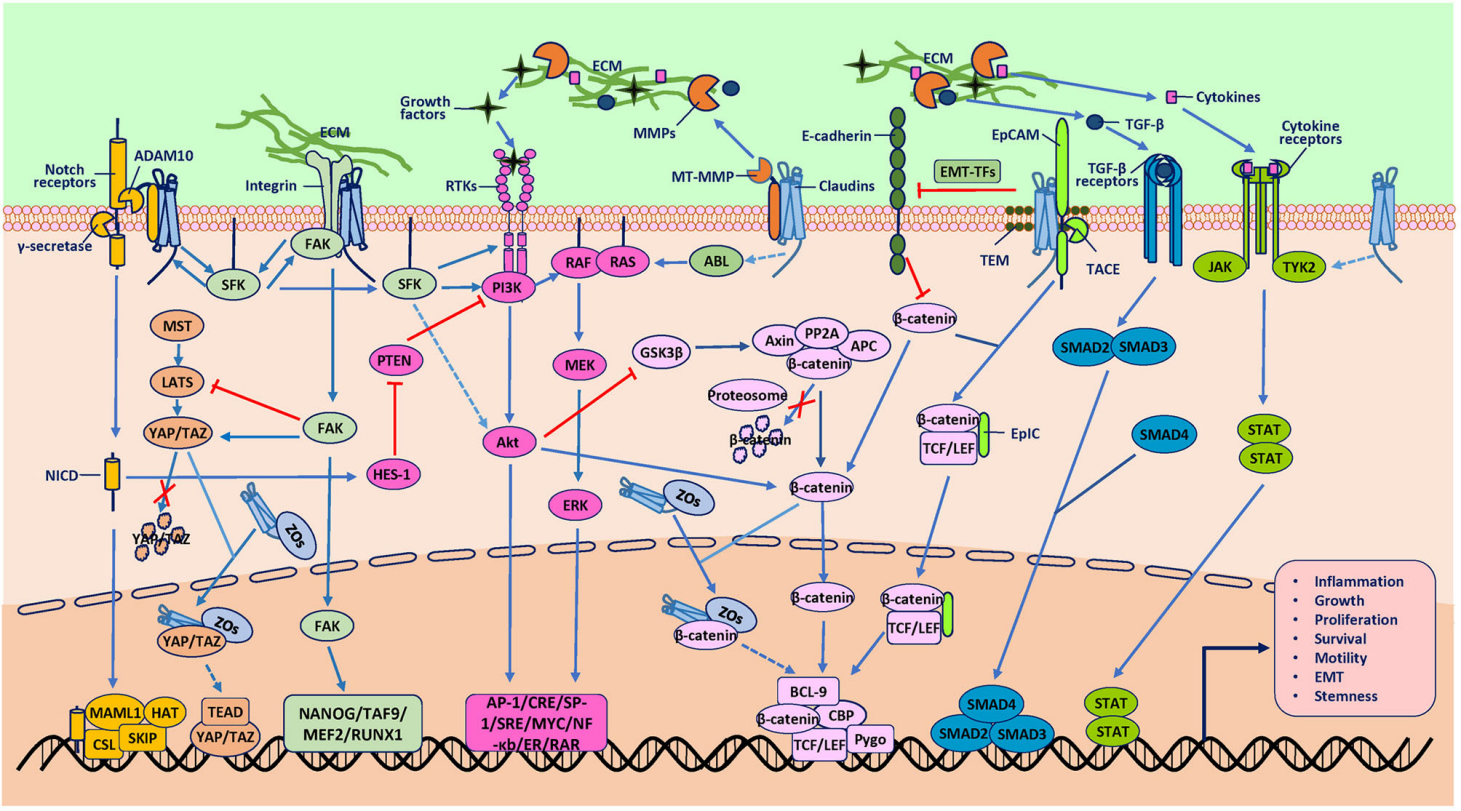
Protumor role of claudins. The mechanisms underlying tumorigenesis due to claudins have also not been well established. Studies mainly support the hypothesis that claudins activate various signaling pathways or proteases to promote tumorigenesis directly and indirectly. One direct pathway involves association with other molecules, such as epithelial cell adhesion molecule (EpCAM), membrane type-matrix metalloproteinases (MT-MMPs), disintegrin and metalloproteinase 10 (ADAM10) and integrins, which activate claudins to participate in signal transduction, extracellular matrix (ECM) degradation and receptor cleavage. Another direct way involves association with transcription factors (TFs) such as YAP/TAZ and β-catenin to induce the nuclear accumulation of these TFs, although the exact mechanisms through which claudins induce gene transcription are not clear. An indirect way in which claudins activate oncogenic pathways is proposed to require two kinds of molecules. One kind is proteases such as MMPs, which cleave the ECM to release GFs to activate the RTK/PI3K, MAPK, TGF-β/SMAD, and JAK/STAT pathways. Another indirect way is through protein kinases, such as SFK, ABL and Tyk2, which can phosphorylate downstream molecules involved in oncogenic pathways, although the mechanisms through which claudins activate these kinases are not clear. In addition, these signaling pathways are extensively interconnected at various levels, which integrate signaling from claudins to promote tumorigenesis. Although there have been studies that reported the effect of claudins on some of the molecules shown, the exact mechanisms involved are not known (represented by dashed line arrows in the figure).

Mechanisms underlying tumorigenesis due to increased expression of claudins have also not been well established. Studies mainly support the hypothesis that claudins activate various signaling pathways or proteases to promote tumorigenesis directly and indirectly (**Figure 2**). In overexpression mouse models, claudin-1 deregulation promoted inflammatory bowel disease (IBD) and colitis-associated cancer (CAC) susceptibility and severity but did not destabilize TJ integrity and permeability, suggesting that protumor functions of claudins are dependent on non-TJ functions (40). Therefore, the protumor claudins have mainly been found to be those that exist outside of TJs. This phenomenon is most obvious for claudin-7, which has a strong basolateral membrane distribution even under physiological conditions (44, 45). At signaling transduction platforms, glycosphingolipid-enriched membrane microdomains (GEMs) or tetraspanin-enriched membrane microdomains (TEMs), claudin-7 recruits epithelial cell adhesion molecule (EpCAM), which is then digested by TNF-α-converting enzyme (TACE) and releases the intracellular fragment EpIC (46). EpIC acts as a cotranscription factor in cooperation with β-catenin and others and contributes to the upregulation of vimentin, Snail, and Slug and the downregulation of E-cadherin (46). Overexpression of claudins can also weaken cell-cell adhesion to induce cancer progression, although the mechanisms are not well explored. Potential mechanisms may involve abnormal localization and suppression of the expression of other adhesion molecules, such as E-cadherin (47). Nuclear claudins are mostly found in malignant tissues, suggesting their role in cancer. They are always found in complexes containing other proteins, such as β-catenin, YAP, ZO-1, ZO-1 nucleic acid-binding protein (ZONAB), and cyclin D1, and induce retention of such molecules in the nucleus to enhance cell proliferation and cancer progression (48–52). The underlying mechanisms contributing to their translocation are not well understood but may involve phosphorylation by protein kinases (53).

In addition to the mechanisms through which claudins have been demonstrated to participate in tumor progression directly, numerous studies have shown that claudins can activate diverse signaling pathways to indirectly promote carcinogenesis (**Supplementary Table S2**). For example, in contrast to the many claudins in TJs that bind to and retain β-catenin in the submembrane compartment to inhibit its activation, claudin-1 was also found to activate β-catenin *via* Notch/Akt signaling to deregulate intestinal epithelial cell (IEC) homeostasis, thus promoting CAC (40). Claudins have also been demonstrated to activate many other signaling pathways and proteases, including phosphoinositide-3-kinase/protein kinase B (PI3K/Akt), mitogen-activated protein kinase (MAPK), Janus kinase/signal transducer and activator of transcription (JAK/STAT), metalloproteinases (MMPs) and microRNAs (miRs), thus contributing to various hallmarks of cancer (**Supplementary Table S2**). However, it is not clear how claudins regulate the activity of the molecules involved in these signaling pathways, and limited studies have been conducted to resolve this question. Perhaps claudins directly activate members of these pathways through their intracellular motifs or interactions in the cytoplasm or nucleus or indirectly activate members of these pathways through interactions with molecules such as β-catenin, ZOs, Src family kinases (SFKs), integrins and EpCAM, which are extensively interconnected at various levels (**Supplementary Table S2**). For example, ECL2-dependent transinteraction of claudin-6 recruits and activates SFK, leading to activation of the PI3K/Akt cascade (54). Another possible mechanism is that claudins have been found to upregulate and/or activate proteases such as MMPs and a disintegrin and metalloproteinase 10 (ADAM10), although the upstream pathways that control this function are not well defined. Matrix component or receptor cleavage by MMPs or ADAM10 results in the release of growth factors, cytokines and intracellular fragments of receptors, such as vascular endothelial growth factor (VEGF), active transforming growth factor-β (TGF-β) and Notch intracellular domain (NICD), that collectively provoke many oncogenic pathways, and these mechanisms need to be validated in future studies (55–57).

### Expansion of Cancer Stem Cells

Specialized claudin signals support the function of normal adult stem cells and their neoplastic derivatives. The crosstalk between claudins and the Hippo/YAP and Wnt/β-catenin signaling pathways, whose functions are important for normal cells and CSCs, has been extensively studied (58). In the intestinal epithelium, claudin-2 expression is restricted to the stem/progenitor cell compartment and plays a regulatory role in intestinal homeostasis (41, 59). The expression of claudin-2 was increased in mouse intestinal tumors displaying elevated expression of CSC markers (60). In human colorectal cancer (CRC), claudin-2 overexpression promoted the self-renewal properties of patient-derived CRC cells and cell lines *in vitro* and *in vivo*, slowed down their differentiation and promoted the phenotypic transition of non-stem cells towards a stem-like phenotype (61). In this study, claudin-2 promoted the self-renewal of CRC stem-like cells through activation of YAP and downstream repression of miR-222-3p (61). Claudin-1 and -2 are known to regulate the β-catenin-T cell factor/lymphoid enhancer-binding factor (TCF/LEF) signaling pathway to regulate CSCs (40, 62). In addition, one of the major contributing factors for the oncogenic role of claudin-3 in non-small-cell lung cancer (NSCLC) is its regulation of cancer stemness (63). The depletion of claudin-3 prevented the formation of spheres and tumor formation (63). Claudin-7 is indispensable for controlling Wnt/β-catenin signaling-dependent intestinal epithelial stem cell survival, self-renewal, and cell differentiation (64).

In contrast, some claudins negatively regulate CSC programs with tissue specificity. Claudin-3 expression was positively associated with colonocyte differentiation. Loss of claudin-3 exacerbated Wnt/β-catenin activation by IL6/gp130/Stat3 signaling, inducing a dedifferentiated phenotype and invasive mobility (25). Claudin-low cancer represents a rare and biologically aggressive variant of epithelial tumor A claudin-low molecular subtype of breast cancer has been described as having low expression of claudin-3, -4, and -7 and E-cadherin, with concomitant stem cell features (65). It had been previously proposed that this breast cancer type originates from undifferentiated stem cells, which was challenged by a recent study indicating that the claudin-low subtype can arise from differentiated epithelial cells that assume a developmental trajectory where they progressively gain stem cell-like characteristics (66). In spite of the debating of its cell of origin, this subtype of high-grade invasive ductal breast carcinoma was found to lack luminal differentiation markers, and its cells most closely resemble mammary epithelial stem cells (65). Consistent with an antitumor role of claudin-18 in lung cancer, loss of claudin-18 has been reported to activate YAP, resulting in increased abundance and proliferation of known distal lung progenitors, alveolar epithelial type II (AT2) cells, and tumorigenesis in mice (27).

In conclusion, it appears that claudins co-opt different signaling pathways to sustain tumor cell self-renewal in a tumor type- and cell of origin-specific manner. It remains to be established whether CSCs are dependent on specific claudin signaling pathways/mechanisms that can be safely targeted therapeutically.

### Cancer Invasion and Metastatic Cascade

**Figure 3:**
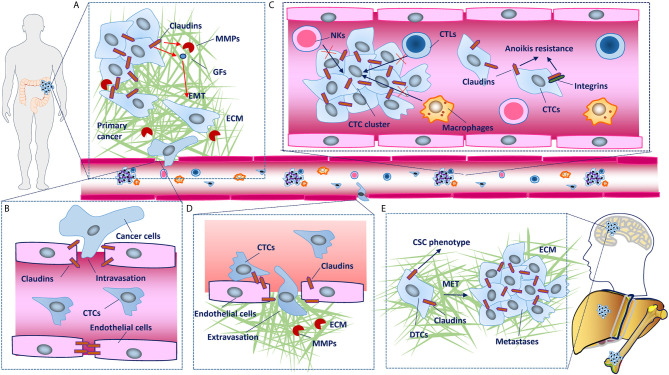
Claudins involved in the cancer invasion and metastasis cascade. **(A)** In primary tumors, disruption of claudin strands in tight junctions (TJs) is necessary for detachment of cancer cells. Some claudins upregulate metalloproteinases (MMPs) and induce epithelial-mesenchymal transition (EMT), which leads to basement membrane degradation, breaching, migration and local invasion. **(B)** Although cancer cells mainly enter the circulation through the lymphatic system, in some conditions, they can also intravasate into blood vessels through disruption of TJs between endothelial cells. **(C)** Claudins facilitate collective migration, which promotes the mutual survival of cancer cells, and claudin dysregulation has also been shown to confer tumor cell resistance to anoikis. **(D)** Claudins on endothelial cells have protective roles against metastasis through barrier function and blood-based interactions with claudins on cancer cells, disruption of which facilitates the extravasation of cancer cells into secondary tissues. **(E)** Establishment of macroscopic metastasis in distant tissues largely depends on the clone forming ability and mesenchymal-epithelial transition (MET) of disseminated tumor cells (DTCs). As chief molecules expressed in epithelial cells that have been demonstrated to maintain the cancer stem cell (CSC) phenotype, claudins inevitably participate in MET and metastatic colonization, although the mechanisms remain to be characterized in detail.

For most solid tumors, the metastatic cascade begins with cancer cells breaching the underlying basement membrane, followed by local invasion and intravasation into the vasculature, survival of circulating tumor cells (CTCs), priming of the metastatic niche, extravasation into the secondary site and metastatic colonization of the new tissue (67). Because patients with solid cancers mostly die of systemic metastatic disease, mechanisms involved in these cascades have been extensively studied (68). Amount of studies have demonstrated that claudins are involved in the cancer invasion-metastasis cascade (**Figure 3**).

#### EMT

EMT has well-established roles in developmental programs involved in generating new tissues and organs, and cancer cells exploit EMT processes to enhance the CSC phenotype and increase metastatic potential (69). Upon activation of the EMT program, cancer cells lose many of their epithelial characteristics, including the presence of epithelial cell junctions and apical-basal polarity (70). As claudins play roles in maintaining these functions and enhancing the CSC phenotype, the involvement of claudins in EMT is not surprising. The claudin-low molecular subtype of breast cancer has concomitant EMT and stem cell features (65, 66). The expression of claudins is regulated by many signaling pathways and transcription factors (TFs), such as Wnt/β-catenin, Slug, and Snail, which are all involved in the EMT program (**Supplementary Table S1**). For example, an E-box element was found in the *CLDN1* promoter, and through this E-box element, Slug and Snail can inhibit the expression of claudin-1 (71). The loss of claudins disrupts the integrity of cell connections, one of the characteristics of EMT. However, many claudins are upregulated in some cancer tissues, and whether and how they are involved in EMT are not well understood. Most studies on EMT have been conducted in traditional cell culture systems, which may not reflect real physiological or pathological conditions well. Evidence has shown that cancer cells that retain more epithelial features with fewer mesenchymal features have the greatest malignant and metastatic potential (72, 73). Therefore, cells with partial EMT may retain some claudin expression. Additionally, claudins act as signaling hubs that regulate many molecules involved in EMT (**Supplementary Table S2**), and this regulation is best understood for claudin-1 in CRC. Claudin-1 activates Wnt and PI3K/Akt signaling, which upregulated the expression of zinc finger E-box binding homeobox 1 (ZEB1) or β-catenin/TCF/LEF transcriptional activity, leading to downregulation of E-cadherin expression (47, 50). In hepatocellular carcinoma cells, claudin-1 was also found to induce the changes in EMT markers (decreased E-cadherin and increased N-cadherin and vimentin expression) by upregulating the expression of the EMT TFs Slug and ZEB1 through the MAPK/extracellular signal-regulated kinase (ERK)1/2 pathways (74).

#### Invasion

The extracellular matrix (ECM) of tumor tissues undergoes profound changes in composition, structure, and mechanical properties compared with that of normal tissues. For most solid tumors, proteolytic activity is required for cancer cells to breach the surrounding ECM, which they accomplish by producing MMPs and other matrix remodeling enzymes (75, 76). The dysregulation of claudins enhances cell invasion *via* the activation of MMPs in certain cancer types. However, no studies have explored the direct mechanisms by which claudins regulate MMPs. Torres-Martínez AC et al. proposed a docking site mechanism in which claudin-1 interacts with extracellular proMMP-2 through its ECLs, thus promoting its activation by MMP-14, which in turn promotes metastatic processes that require increased cell migration and invasiveness (77). Claudin regulation of the expression and activity of MMPs is another plausible mechanism. For example, claudin-1 was found to activate MMP-2 through the activation of the c-Abl-protein kinase Cδ (PKCδ) signaling pathway, while increased secretion of MMP-2 correlated with cytoplasmic expression of claudin-1 in metastatic melanoma cells in a PKC-dependent manner (78–80). In A549 cells, claudin-2 elevated the nuclear levels of Sp1, which increased the mRNA level and enzymatic activity of MMP-9 (81).

To date, studies on the contribution of proteases to tumor invasion have mainly focused on their role in ECM degradation. On the other hand, it is becoming increasingly evident that proteases also promote a highly invasive phenotype through cleavage of cell-cell adhesion molecules to free cancer cells from their neighbors (82). However, most studies have focused on adhesion molecules such as E-cadherin, and data supporting the function of proteases in cleaving claudins are limited (83). Trypsin-2 (Try2), a tumor-associated serine proteinase, has been shown to activate pro-membrane type 1 MMP (MT1-MMP), leading to downregulation of claudin-7 and disruption of TJs, which induce tongue carcinoma invasion (84). Although in this study, the authors indeed demonstrated that claudin-7 can be cleaved into smaller fragments by MT1-MMP that *in vitro*, whether claudins integrated into TJs are also cleaved by these extracellular proteases remains unclear (84).

#### Migration

In addition to invasion, increased cell motility is also an important contributor to tumor progression. Local cell migration and dissemination allow tumors to expand and reach higher growth rates; otherwise, tumor cells will lose their rapid growth capacity owing to crowding and contact inhibition (85). Cancer cell migration involves complex cell-cell and cell-ECM contacts, and changes in the expression or localization of claudins affect many aspects of this program. Disruption of TJs by claudin dysregulation induces detachment of cancer cells and then activates or inhibits many oncogenic pathways that play a critical role in the movement of cancer cells, and these functions differ based on the tumor type and the state of the disease. For example, claudin-18 was shown to suppress the abnormal motility of lung epithelial cells by inhibiting the PI3K/PDK1/Akt signaling pathway, while claudin-6 promoted cell migration through activation of the same signaling pathway (35, 86). Claudin-1 overexpression brings morphological changes and increases the intercellular adhesion through the disappearance of stress fibers, resulting in the migration inhibition of breast cancer mesenchymal-stem-like subtype cells (87). However, claudin-1 has also been found to participate in cancer cell migration through activation of focal adhesion kinase (FAK), another nonreceptor protein tyrosine kinase that controls fundamental cellular processes, including migration under physiological or disease conditions (88). Numerous studies have linked EMT, loss of E-cadherin-mediated adhesion junctions (AJs) and increased integrin-mediated adhesions to the dissemination of individual cancer cells (67). In addition to the important roles claudins play in EMT and the expression of E-cadherin, claudins also interact with integrins to control cell-ECM contacts. Several claudins have been found to interact or form complexes with integrins (with such complexes mainly involving claudin-7 and integrin β1) to mediate cancer cell migration through activation of FAK, cytoskeleton reorganization and ECM alteration (38, 89–91).

Various modes of cell migration, ranging from single-cell motility to collective cell migration as sheets or strands, are observed *in vivo* (92). However, disseminated individual cancer cells are rarely detected in human cancers; instead, collective cell migration in which cells remain associated as they move is frequently observed at the invasive front of tumors and clustering CTCs (93–95). It has been postulated that upregulation of claudin-11 involved in cell-cell contacts prompts collective migration in squamous cell carcinoma, and this process was also regulated by EMT-inducing TFs such as Snail (96). In head and neck cancer patients, claudin-11 prompts the formation of CTC clusters, which correlates with poor prognosis (96). Claudin-1 has also been shown to facilitate the collective migration of cancer cells in several tumor types (97, 98). In addition, cancer cells may induce claudin-11 overexpression and subsequent collective migration of peritumoral carcinoma−associated fibroblasts (CAFs) *via* TGF-β secretion (99). The mechanisms by which claudins promote the collective migration of cancer cells are not fully understood. It appears, however, that some claudins may retain the TJs between cancer cells, and moreover, claudins can form complexes with integrins to regulate cell-cell and cell-ECM interactions. The balance between these interactions may determine the migratory model (67).

#### Intravascular Survival

Epithelial cells normally undergo apoptosis after they lose contact with their ECM or neighboring cells (100). Survival from anoikis and the harsh environment tumor cells encounter once they leave the primary site and exist in the bloodstream for long enough to home to a distant site that permits dissemination is a major challenge and possibly the most critical step for distant metastasis. In addition to prompt collective migration, which promotes mutual survival, claudin dysregulation has also been shown to confer tumor cell resistance to anoikis. For example, claudin-1 and claudin-2 modulate anoikis to induce cancer progression in a Src-Akt-Bcl-2- and epithelial growth factor receptor (EGFR)-dependent manner, respectively, while loss of claudin-18.1 increases anchorage-independent colony formation *in vitro* in lung cancer cells through activation of YAP/TAZ, insulin-like growth factor receptor-1 (IGF-1R) and AKT signaling (35, 36, 101, 102). Integrins are indispensable in cancer cell migration and promote anchorage-independent survival through various mechanisms; therefore, claudins may be indirectly involved in the anoikis resistance of cancer cells through integrins (67). However, no studies to test this effect of claudins have been conducted.

#### Intravasation, Extravasation and MET

There have been limited studies exploring the roles claudins play in other metastatic pathways. However, one can speculate that claudins are involved in these events. For example, claudins also participate in the establishment of vascular endothelial sheets, and cancer cell disruption of the barrier function of claudins is important for their intravasation into the vasculature and extravasation into secondary sites, especially in the brain, which is protected by the unique properties of the blood-brain barrier (BBB). One study reported that claudin-5 regulates the permeability of the BBB by regulating the proliferation, migration, and permeability of hCMEC/D3 cells, especially by modulating the cell adhesion molecule signaling pathway, to enhance the function of TJs, which are involved in reducing the formation of lung cancer brain metastasis (103). Claudin-1 has also been found to mediate interactions between cancer cells and brain endothelial cells, such as those occurring in TJs, and consequently inhibit transmigration (104). Therefore, disruption of these claudins is an essential step for successful extravasation to form brain metastases. Several mechanisms have been demonstrated to be utilized by cancer cells to disturb the interendothelial barriers. MMP-1 secreted by breast cancer cells disrupts interendothelial junction complex molecules, including claudin-5, allowing cancer cells to transmigrate through the brain endothelial barrier to form brain metastases (105). In response to proinflammatory stimuli, endothelial Tumor progression locus 2 (Tpl2) alters tight junction claudin-5 protein expression, resulting in increased vascular permeability, immune cell infiltration and metastatic diseases (106). Generating a macroscopic metastasis in distant tissues, which is important for the final step of malignant progression, largely depends on mesenchymal–epithelial transition (MET), the reverse of EMT. MET is characterized by repression of mesenchymal traits and re-expression of epithelial markers, ultimately resulting in the formation of metastatic colonies that contain hierarchically organized cells resembling the corresponding primary tumors (69). As chief molecules that are expressed in epithelial cells, claudins inevitably participate in MET, although their roles remain to be characterized in detail.

### Claudins in Chemoresistance

EMT and CSC functions are two vital mechanisms responsible for the chemoresistance of cancer cells. Since the role of claudins in the regulation of EMT and CSCs is well documented, their correlation with drug resistance is inevitable and obvious (107, 108). A potential role of claudin-6 in enhancing chemoresistance to adriamycin in triple-negative breast cancer (TNBC) was documented, which was mediated through the upregulation of CSCs (109). Claudin-3-overexpressing lung cancer cells were insensitive to cisplatin treatment, and targeting transcription with small molecules suppressed cancer stemness and reversed chemoresistance (63). Although the tumor-promoting role of claudins is achieved through loss of expression or increased expression outside TJs, studies have reported that tumor cells retain claudins localized within TJs, where they lead to a decrease in paracellular permeability and inhibit the penetration of anticancer drugs into the inner area of spheroids (110). In addition, dysregulated claudins can induce chemoresistance through other mechanisms, such as transport and autophagy. For example, knockdown of claudin-3 or claudin-4 in ovarian cancer cells induced resistance to cisplatin by regulating the Cu transporter CTR1 (111). In lung cancer, claudin-1 is a key factor responsible for resistance to cisplatin, which is accomplished by activating autophagy *via* the upregulation of Unc-51-like autophagy activating kinase 1 (ULK1) phosphorylation (112). Moreover, claudins may regulate sensitivity to different antitumor drugs through different mechanisms. Claudins participate in a complex network requiring interactions with cytoskeleton factors, including actin and microtubules, through adaptor proteins that contain multiple protein-protein interaction motifs (10). In ovarian tumor cells, claudin-4 was found to interact with both α-tubulin and β-tubulin, affecting the structure and polymerization of the microtubule network and leading to a reduced apoptotic response to paclitaxel, a microtubule-targeting drug. Therefore, inhibiting claudin-4 activity with a claudin mimic peptide (CMP) enhanced the apoptotic response to paclitaxel but not that to cisplatin (113).

## The Complexity of Claudins in Cancer

**Figure 4:**
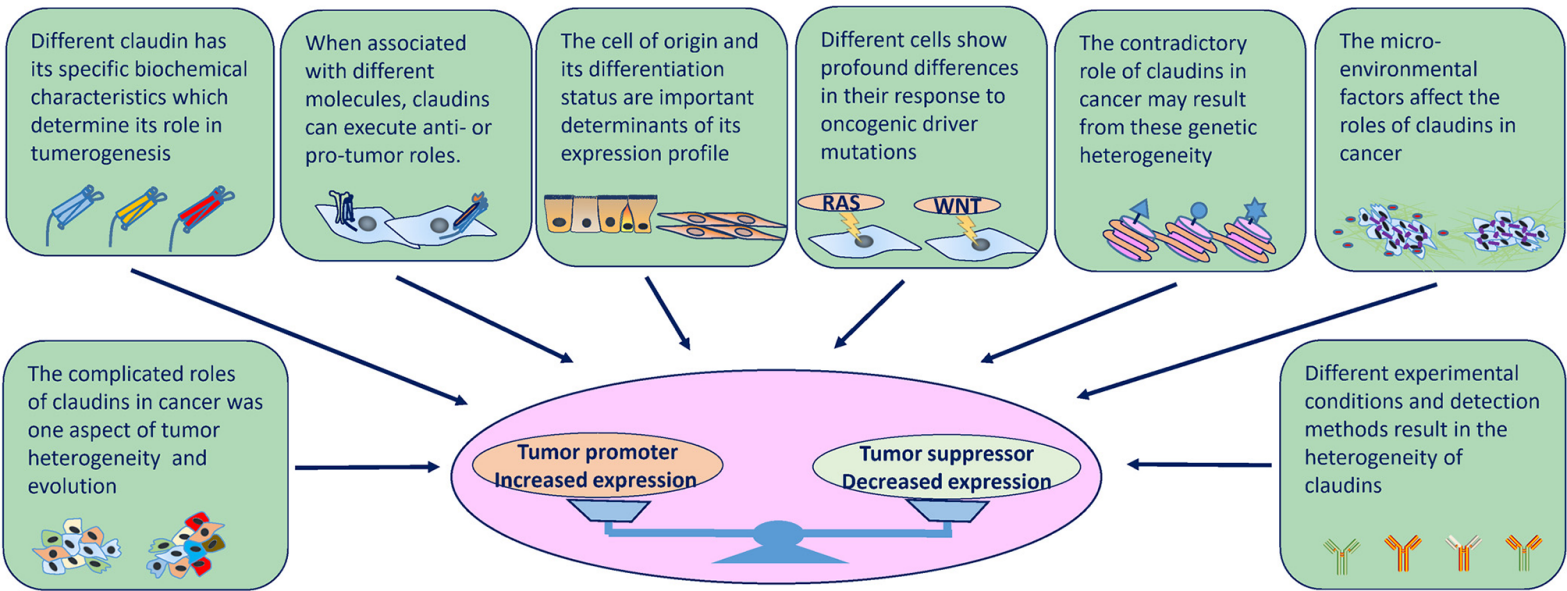
Factors that influence the expression and role of claudins in tumorigenesis at several levels. Intrinsic factors (molecular structures and physiology roles) and extrinsic factors (cell of origin, genetic background, tumor heterogeneity, cancer evolution, TME and environmental factors, experimental conditions and detection methods) determine the divergent expression patterns and roles in cancer.

As mentioned above, the roles of claudins in cancer are heterogenous both within the same cancer type and across different cancers. These discrepancies can be explained on several levels by different factors. Intrinsic factors, including the diversity of claudin molecular structures and the inherent roles claudins play in cell physiology, and extrinsic factors, including the cancer cell of origin, genetic background and evolutionary path, as well as the pre-existing or acquired tumor microenvironment (TME) and environmental factors, determine the divergent expression patterns and roles in cancer (**Figure 4**).

### The Distinction of Individual Claudins

Each claudin has its own characteristics that are determined by its specific amino acid sequence, and the ECL sequences are poorly conserved among claudins. The different amino acid sequences and electrostatic potentials determine the barrier function and charge selectivity (8). Therefore, dysregulation of different claudins may cause different or even opposite outcomes. For instance, in the hepatobiliary system of mouse models, KO of claudin-2, a paracellular “channel-forming” claudin, leads to a decrease in paracellular water transport, while KO of claudin-3, a paracellular “barrier-forming” claudin, leads to an increase in paracellular water and phosphate ion transport (114–116). Furthermore, inflammation, increased cell proliferation and tumors only develop in mice with KO of specific paracellular barrier-forming claudins, whereas mice with KO of specific paracellular channel-forming claudins mostly show absorption and secretory disorders such as decreased bile flow and kidney stone disease (7, 117). In living organs, the scenario is more complicated; several claudins are expressed in the same tissue simultaneously and dynamically, and different combinations and ratios of these proteins confer different permeability and barrier characteristics (12, 118, 119). However, current studies mostly focus on a single claudin, the dysregulation of which leads to compensation by or secondary disorders of other claudins. For example, overexpressed claudin-5 increases claudin-5:claudin-18 interactions while decreasing ZO-1:claudin-18 colocalization to destabilize TJs, in turn increasing paracellular leakage (120). Similar interactions also occur between claudin-4 and claudin-8 and between claudin-16 and claudin-19, although the outcomes and affected tissues are different (121, 122). However, these data are derived from noncancerous conditions, and no evidence is available to support such interactions in cancer. Because the co-dysregulation of claudins is common in cancer, one can infer that complicated interplay between claudins is inevitable.

### Different Locations and Molecular Interactions of Claudins

The different locations and molecular interactions of claudins also lead to their functional heterogeneity. In TJs, claudins form highly organized membrane complexes and usually drive contact inhibition by sequestering YAP away from the nucleus (123). In the context of cancer cells, where TJs and contact inhibition are at least partially lost, overexpression of claudin-2 enhances YAP activity in coordination with other oncogenic signaling networks (124). When binding with EpCAM, claudin-7 promotes cancer progression, while when binding with integrin β1, claudin-7 maintains cell-matrix adhesion and suppresses the growth and movement of cancer cells (38, 46, 90). However, despite claudin-2 being a “channel-forming” claudin, when claudin-2 and claudin-4 are not integrated into TJs, they serve as ligands for or increase the expression of integrin β1, activate FAK and promote stemness and metastasis (61, 91, 125). Moreover, even when interacting with the same molecule within the same cancer context, individual claudins may also display dual effects. For example, in lung cancer cells, claudin-7 suppresses cancer cell proliferation and increases the expression of and interacts with integrin β1 to maintain epithelial cell attachment, and these cell-matrix interactions also support cell migration and invasiveness (38, 89). These results indicate that claudin-7 acts as a tumor suppressor in primary tumor initiation but facilitates cancer progression in metastatic−stage disease. In addition, some functions related to specific claudins remain to be discovered. For example, although previous studies reported that mice lacking the stomach isoform of claudin-18 showed increased paracellular permeability, which accounted for the development of inflammation and gastric tumors, the findings of a recent study suggest that claudin-18 KO has no effect on paracellular permeability and rather affects anion permeability; these effects may be secondary to transcellular anion transporter expression/function changes in the absence of claudin-18 (34, 126, 127). Further experiments should be performed to examine yet undiscovered relationships between transcellular transport and claudins.

### Cell of Origin and Differentiation Status

In cancer, the cell of origin and differentiation status are important determinants of the tumor expression profile, which is shaped during the development of the tumor, although alterations always occur to some extent after malignant transformation. For example, although claudin-1 is expressed in a large number of tissues, its role is especially crucial for squamous epithelium such as skin, whose germline mutations mainly cause neonatal ichthyosis. Therefore, in lung cancers, high claudin-1 expression is more likely to be observed in tumors with a squamous cell phenotype than in tumors with other cell phenotypes, whereas tumors with adenocarcinoma components are likely to lose claudin-1 expression (128). The expression of *CLDN1* was high in the TCGA CESC, HNSC, ESCA, LUSC, CHOL and THCA datasets, most of which are squamous cancers. Another example is claudin-6, which is an oncofoetal cell surface antigen that is completely silenced in normal human tissues but reactivated in germline tumors such as testicular, ovarian and uterine cancer (129).

Another mechanism contributing to the different roles played by claudins according to cell of origin is tissue-specific expression. Studies have shown that claudin-1 is expressed by brain endothelial cells but not by peripheral lung parenchyma endothelial cells (128, 130). Similarly, claudin-3, which forms heterophilic interactions with claudin-1, is also expressed by brain endothelial cells but not by lung endothelial cells (130, 131). Therefore, when cancer cells induce disruption of the junctions between endothelial cells, claudin-1 is exposed to the circulation; transmigration of cancer cell-expressed claudin-1 can be inhibited by homotypic claudin-1 interaction, which suppresses brain metastasis. In contrast, cancer cells can induce transmigration of endothelial cells to successfully metastasize when claudin-1 is lost on circulating lung endothelial cells (104).

Moreover, key studies have shown that stem and progenitor cells in normal tissues are particularly susceptible to oncogenic transformation, and transformation includes a reversal of the differentiation signals put in place during development; thus, when benign lesions transition into malignant lesions, they undergo progressive acquisition of characteristics of the undifferentiated state (132). In this regard, some cancer types display a hierarchical nature founded by a CSC or a malignant cell that has gained stem cell properties (133). This phenomenon may explain the dysregulation of claudins in some cancers. For example, as discussed above, claudin-2 expression is restricted to the stem/progenitor cell compartment in the normal intestinal epithelium; however, the expression of claudin-2 is increased in human CRC (41, 59, 61). In contrast, the expression of claudin-18.2 is restricted to differentiated gastric cells and absent from the stem cell zone of gastric glands (134). When gastric cancer arises from these stem cells or the cells transition into an undifferentiated state, the expression of claudin-18.2 significantly decreases (134).

### Genomic Alteration Diversity

Currently, cancer is regarded as a collection of diseases characterized by uncontrolled cellular growth caused primarily by genomic (here referring to both genetic and epigenetic) alterations. Mutations occur in a set of genes called ‘cancer driver genes’, conferring somatic cells with certain selective advantages over neighboring cells (135). In addition, after malignant transformation, a cancer remains dynamic and continues to evolve, leading to molecular heterogeneity. As summarized in **Supplementary Table S1**, the frequencies and types of genetic alterations of *CLDNs* in tumors differ depending on the cancer and *CLDN* type, and an important degree of variability across cancer subtypes (histological and molecular) is also observed. Some alterations occur across several cancer types, while others tend to be more specific. Therefore, the contradictory roles of claudins in cancer may result from this genetic heterogeneity. A prime example of this is the expression characteristics of claudin-1, which is always overexpressed in squamous cancer types, consistent with its high amplification rates in lung squamous, oesophageal, cervical, and head and neck cancers. Another claudin gene, *CLDN16*, is highly amplified and expressed in ovarian cancer. However, in many cases, the impact of copy number alterations on the expression of an individual gene is relatively subtle, and inconsistencies between amplification and expression levels are also observed (136). The amplification frequencies of *CLDN11* and *CLDN16* are high in lung squamous, oesophageal, cervical, and head and neck cancers, but the expression levels are low in these cancer types. In addition, although deep deletion of *CLDN20-25* is a common event in cancers, it has not been validated at the protein level; therefore, the role of deep deletion of these genes in cancer is not known. Adding yet another level of complexity, the epigenetic state, reflected in context-specific differences in chromatin organization and DNA accessibility, may also direct the level of claudins in cancer (137, 138). In CRC and hepatocellular carcinoma (HCC), promoter hypermethylation of *CLDN3* is correlated with decreased claudin-3 expression, but promoter derepression leads to upregulation in prostate and ovarian cancers (25, 139, 140). In line with these findings, siRNA silencing of the DNA methyltransferase DNMT-1 differentially affects claudin-3 expression in PC3 (prostate cancer cells) and Caco-2 cells, suggesting tissue-specific regulation (25, 141). Epigenetic mechanisms have a greater impact on tumor cell phenotypes than genetic alterations (142), and the epigenetic status of tumor cells can be highly plastic, suggesting that epigenetic changes have a dominant impact in shaping claudins expression, consistent with the diverse and dynamic expression patterns of claudins.

The mechanisms that contribute to the distinct but diverse genomic alterations of *CLDNs* are not clear. Genomic alterations may play a driver role in tumorigenesis and undergo positive selection. However, there is no definite evidence to support this role in human tumorigenesis, although many genetic manipulation and mouse model experiments have shown that claudin dysregulation contributes to many hallmarks of tumors. In addition, these alterations may be passengers rather than drivers but under the influence of other driver alterations; some of them could have no involvement at all in tumorigenesis, while some of them may aid in tumor initiation and/or progression. For example, missense mutations of claudin genes are unusual in cancer, and no mutation clusters have been found along the sequence of these genes. However, the missense mutations that have been found mostly occur in uterine cancer and melanoma, two cancer types with highly mutated genetic backgrounds (143). The only recurrent genomic rearrangement involving interchromosomal translocation between claudin-18 and Rho GTPase activating protein 26 (CLDN18-ARHGAP26) was found in the genomically stable (GS) subtype of gastric cancer; this alteration is also enriched in the diffuse type and mutually exclusive with RHOA mutations. The generated fusion protein has been speculated to affect ARHGAP’s regulation of RHOA, cell motility and cellular adhesion, which contribute to the invasive phenotype of diffuse gastric cancer (143, 144). However, why this fusion event only occurs in this specific context is not clear currently; both mechanisms explained above may be possible explanations.

### Context-Specific Oncogenic Pathway Function

In cancer cells, oncogene activation lies upstream of most protein expression dysregulation in the majority of instances. However, although cellular responses and cell fate decisions are controlled by a limited number of signal transduction pathways and dysregulation of any one of them contributes to tumorigenesis, different cell and tissue types show profound differences in their response to oncogenic driver mutations (145). In addition, the organization of oncogenic signaling pathways and the output of an oncogenic driver can differ substantially between tissue types, which leads to different effects on claudins. For example, in lung cancer cells, claudin-1 is upregulated through activation of the PI3K/Akt/NF-κB pathway, decreasing permeability and resulting in inhibition of the penetration of anticancer drugs into the inner area of spheroids (110). In pancreatic cancer, PKCα is highly expressed, and cancer cells are addicted to PKCα. PKCα downregulates claudin-1 *via* Snail- and MAPK/ERK-dependent pathways, which leads to decreased cell-cell adhesion during EMT in pancreatic cancer (146). Moreover, the membrane zinc importer ZIP4 was found to be overexpressed in human pancreatic cancer and promotes tumor growth and metastasis, and it was demonstrated that ZIP4 represses claudin-1 through a ZEB1-dependent transcriptional mechanism, which leads to activation of FAK and paxillin to cause increased cell migration, invasion and tumor metastasis (147, 148). The contradictory roles of claudin-1 in different cancers reflect that different oncogenic pathways can hijack different roles of claudins to contribute to cancer progression.

On the other hand, claudins also contribute to cancer development *via* different activated oncogenic pathways in a context-dependent manner. Claudin-1 expression is significantly increased in CRC subtypes associated with marked Wnt signaling activation, such as the Marisa C5, Sadanandam TA and CMS2 consensus subtypes (149). In mouse models, claudin-1 overexpression obviously increases the activity of Wnt and Notch signaling, leading to the proliferation of tumor cells (39). These findings are in accordance with the already described involvement of Wnt signaling in CRC (150).

### Microenvironmental Heterogeneity

Tumor formation not only entails genetic and epigenetic transformation of normal cells but also needs and contributes to highly aberrant microenvironments. In addition, signaling pathways not only transmit but also encode, process and integrate external and internal signals, providing a specific and appropriate response to external stimuli. Therefore, the output of these integration signals on claudins is further complicated by the presence and combinatorial action of other factors, such as spatial and temporal variability in nutrients, oxygenation, growth factors, and hormones. For example, oestrogen/GPR30 signaling induces claudin-1 expression in estrogen receptor (ER)-negative cervical adenocarcinoma (151). In breast cancer, claudin-1 acts as a tumor suppressor in ER-positive cancer and as a tumor promoter in ER-negative cancer (152). Glucose was found to regulate the expression of claudin-2 in endometrial cancer, affecting proliferation, migration, and invasion (153). Additionally, some vitamins can regulate the expression of claudins. For example, vitamin D can regulate claudin-2, -4 and -15 protein levels, suggesting that vitamin D administration to ulcerative colitis (UC) patients could be a useful therapeutic intervention to relieve intestinal inflammation and prevent CAC (154, 155).

### Tumoral Heterogeneity and Detection Methods

Although some of the above findings were obtained from studies on human tissues, most were based on genetic manipulation in cells or animal models; as such, the limitations are obvious, and these data may not reliably reflect the actual situation in human tumors. Opposite results can be obtained for specific claudins in the same tumor but under different experimental conditions. In addition, at the human tissue level, heterogeneity is also inevitable and can result from genetic, transcriptomic, epigenetic, and/or phenotypic changes. The expression patterns and prognostic roles of claudins in human cancers are diverse and inconsistent, with cancer type- and stage-specific patterns (43). With regard to tumor heterogeneity, many excellent reviews have been published, and readers can reference them (136, 156). At the population level, claudin heterogeneity may result from challenges in acquiring patient samples at equivalent stages, and in classifying claudin expression based on different methodologies and cutoffs. Therefore, the reported claudin expression patterns and fluctuation of claudin levels at different stages of disease progression may be dynamic properties of the disease. Altered claudin expression, although evident in many cancers, may be a passenger and may not contribute or participate in tumorigenesis; therefore, claudins may not be the defining feature of the disease or might only assist in the initiation and progression of cancer under the control of driver oncogenic pathways.

Overall, stratification based on tumor subtype, stage and heterogeneity on the basis of claudin isoform profiles should be carried out to delineate claudin function in cancer in future studies. Additionally, it is important to take various aspects, including the organ type, environmental context and genetic confounders, into account when studying the biological function and targeting of claudins in cancer.

## Future Perspectives and Conclusion

Our understanding of the role of claudins in cancer is incomplete, and exploration is still lacking in some areas of cancer biology. For example, mechanical cell competition is induced by differential sensitivity to cell crowding and causes cells that are more sensitive to crowding to be eliminated by cells with less sensitivity to crowding. This phenomenon can promotes tumor progression when tumor cells behave as winners or suppress tumor progression when normal cells are the winners (157, 158). Claudins are molecules that mediate intercellular interactions, and studies have shown that claudin-based TJs participate in a complex protein network that interacts with the cytoskeleton and can respond to tensile forces, in addition to having barrier and signaling functions, as discussed above (10, 159). Whether claudins play a role in regulating epithelial homeostasis *via* cell density sensing and whether claudin dysregulation contributes to tumor cell competition are worthy of further research. Another example is asymmetric division in stem cells, a process through which posttranslational modifications (PTMs) can cause differential segregation and inheritance of proteins occur during cell division, which is critical for normal tissue homeostasis and diversification. Switching of asymmetric division to symmetric division (renewal) as a result of oncogenic events leads to the expansion of CSCs, which subsequently drive a more aggressive, undifferentiated state (132). Although our understanding of the mechanisms controlling asymmetric division is still in its infancy, the PAR3-PAR6-aPKC complex has been shown to play an important role in this program (160). TJ proteins act as scaffolds for polarity signaling proteins, including the PAR-3/PAR-6/aPKC complex, playing important roles in epithelial polarity (161–164). Whether dysregulation of claudins and TJs contributes to misappropriation of asymmetric division in CSCs needs further study. The TME comprises all the nonmalignant host cellular and noncellular components of the tumour niche, including, but not limited to, the ECM, fibroblast, vascular cells and immune cells. The importance of the TME in the development, growth, and progression of cancer is now well recognized. As cell surface molecules, interactions between claudins and components of TME is inevitable. Although claudins are mainly found to form TJ through interaction with the same claudins and claudins are rarely expressed in cells except for epithelial and endothelial cells, interactions between claudins and other molecules to facilitate tumor progression have also been reported (165, 166). However, interactions between claudins and other constituents of TME, especially the immune cells, such as whether the aberrant expression of claudins have any immunogenicity and the effects on immunotherapy, needs further investigation.

Despite the complexity of claudins in cancer, from a translational perspective, although normal epithelial cells also express claudins, claudin expression has tissue specificity, and some claudins are only expressed in very few tissue types (10, 167, 168). In addition, it has been observed that most claudins, if not all, are buried in the TJ complex in normal tissues, while in malignant tissues, higher accessibility of claudins is caused by extrajunctional mislocalization of the molecules (169–172). Owing to this specific expression profile and difference between normal and tumor cells, claudins are attractive targets that can theoretically enable selective drug delivery with minimal adverse events. Therefore, many potential approaches for targeting claudins in patients with cancer are available, which include Clostridium perfringens enterotoxin (CPE), monoclonal antibodies (mAbs), C-terminal of CPE (C-CPE) or mAb-drug/material conjugates, bispecific T cell engagers (BiTEs) and chimeric antigen receptor (CAR) T cells (129, 173–176). Claudin-targeting strategies seem to hold substantial promise, and this idea has been validated by clinical applications using specific claudin-targeting therapies (targeting claudin-6 and claudin-18.2) (177). However, although proof-of-concept experiments have verified the antitumor effect of many other claudin-targeting therapies, most of them remain in the laboratory stage, and their translation into clinical practice is eagerly awaited.

Fundamentally, alteration of claudins is a common phenotype associated with many different cancer types. Although they were initially found to be an essential component of TJs initially, cancer type- and stage-dependent antitumor and protumor functions have been well established. Indeed, as our understanding of the mechanisms by which claudins participate in tumorigenesis increases, it is likely that we will find that many of the effects of claudins are in fact composites of various tumor-promoting and tumor-suppressing signaling pathways. Moreover, an improved understanding of the integration and functionality of specific claudins within these signaling pathways in different cancer types, stages and microenvironments in malignant progression will be crucial for understanding their role in tumorigenesis and thus in cancer treatment and prevention.

## Author Contributions

The manuscript including tables and figures was drafted and revised by JL. The author confirms being the sole contributor of this work and has approved it for publication.

## Conflict of Interest

The author declares that the research was conducted in the absence of any commercial or financial relationships that could be construed as a potential conflict of interest.
