# Supplementary material for: Context-Dependent Roles of Claudins in Tumorigenesis
Source: Front Oncol. 2021 Jul 20;11:676781. doi: 10.3389/fonc.2021.676781 (PMC8329526; doi:10.3389/fonc.2021.676781)
Supplement: Supplementary file 1 [file Table_1.docx]

Supplementary Material

**Supplementary Table S1. Expression regulation of claudins in cancer**

| Claudins regulation | Regulator | Cancer type | Mechanism | Function |
| --- | --- | --- | --- | --- |
| Epigenetic | DNA methylation | Breast cancer | DOCK1→RRP1B→DNMT→claudin-1↓ | Viability and motility↑(1) |
|  |  | Breast cancer | TGF-β→SMAD2→DNMT1→claudin-6↓ | EMT, migration and invasion↑(2) |
|  | Histone modifications | HCC | EZH2→H3K27ME3↑→claudin-14↓→Wnt/β-catenin signaling activity↑ | Motility, invasive and EMT↑(3) |
|  | Chromatin structure reconfiguration | Lung cancer | Spi-B→chromatin structure reconfiguration→ claudin-2↓ | Invasiveness↑(4) |
| Signaling pathways and TFs | ERK, Akt and/or β-catenin | Breast cancer | ADAM15→PI3K/Akt/mTOR↑→claudin-1↑ | Cell clustering↑(5) |
|  |  | Lung cancer | PI3K/Akt/NF-κB→claudin-1↑ | Anticancer drugs penetration↓(6) |
|  |  | LUSC | PDK1→Akt→?→claudin-1 and claudin-11↑ | Anticancer drug-induced toxicity↓(7) |
|  |  | Cervical cancer | Estrogen→GPR30→ERK and/or Akt↑→claudin-1↑ | Survival, proliferation, migration and invasion↑(8) |
|  |  | Pancreatic cancer | PKCα→Snail and MAPK/ERK↑→claudin-1↓ | EMT↑(9) |
|  |  | CRC | β-catenin→claudin-7↓ | EMT↑(10) |
|  |  | Breast cancer | PI3K/Akt→β-catenin↑→claudin-1↑ | Migration and invasion↑(11) |
|  |  | CRC | EGF→ERK1/2 and PI3K/Akt↑→claudin-3↑ | Migration and anchorage-independent colonies↑(12) |
|  | TGF-β/SMAD | Lung cancer | TGF-β→Smad→claudin-4↑ | Motility and tumorigenicity↑(13) |
|  | JAK/STAT | HCC | hGH→STAT3→claudin-1↓ | Invasive and CSC-like properties↑(14) |
|  | EMT-TFs | SCC | Snail→claudin-11↑ | Collective cell migration and invasion↑(15) |
|  |  | Pancreatic cancer | ZIP4→ZEB1→claudin-1↓→FAK and Paxillin↑ | Migration and invasion↑(16) |
|  | Others | Gastric cancer | RUNX3→claudin-1↑ | Tumorigenicity↓(17) |
|  |  | Prostate cancer | AR→claudin-8↑ | Proliferation and migration↑(18) |
|  |  | Gastric cancer | NSAIDs→intracellular Ca2+↑→claudin-4↑ | Anchorage-independent growth and cell migration↓(19) |
|  |  | Lung cancer | TNF-α→PKCδ/iPLA2/PGE2/PPARγ↑→claudin-1↑ | Morphology changes and migration↑(20) |
| mRNA stability | miRs | CRC | miR-98 →claudin-1↓→Bax and RUNX3↑; Bcl-2, C-myc and PCNA↓ | Apoptosis and G1 arrest↑; proliferation, migration and invasion↓(21) |
|  |  | Cervical cancer | miR-1193→claudin-7↓ | Proliferation, invasion and migration↓(22) |
|  |  | Retinoblastoma | miR-361-5p→claudin-8↓ | Proliferation↓, apoptosis↑(23) |
|  |  | HCC | miR-486→claudin-10↓ | Growth, colony formation and migration↓(24) |
|  |  | Gastric cancer | miR-421→claudin-11↓ | Proliferation, invasion and metastasis↑(25) |
|  |  | Lung cancer | miR-146-5p→claudin-12↓→Wnt/β-catenin and PI3K/AKT/MAPK↑ | Cell viability, migration and invasion↑; apoptosis↓(26) |
|  |  | Gastric cancer | miR-1303→claudin-18↓ | Proliferation, migration and invasion↑(27) |
|  | LncRNA | Colon cancer | LINC00662→miR-340-5p↓→claudin-8/IL22/ERK↑ | Proliferation, invasion and migration↑; apoptosis↓(28) |
|  |  | Gastric cancer | LncRNA PCAT18→miR-135b↓→claudin-11↑ | Proliferation, migration and invasion↓(29) |
| PTMs | Phospharylation | SCC | Claudin-11 tyrosine phosphorylation→Src↑→ p190RhoGAP↑→ RhoA activity↓→stable cell-cell contacts↑ | Collective cell migration and invasion↑(15) |
|  |  | RCC | EphA2/Ephrin A1→claudin-4 tyrosine phosphorylation↑→cytoplasmic translocation↑;  PKC-ε→claudin-4 serine phosphorylation↑+YAP→nuclear translocation↑ | EMT↑(30) |
|  |  | Melanoma | PKA→claudin-1 phosphorylation↑→nuclear localization | Metastatic capacity↑(31) |
|  |  | Ovarian cancer | PKCepsilon and PKA→claudin-3 and claudin-4 phosphorylation↑→dislocalization | TJ strength↓(32, 33) |
|  | Palmitoylation | RCC | Palmitoylated claudin-7→GEM→integrin and EpCAM recruitment↑→cytoskeletal linker proteins and MMP14, CD147 and TACE association↑ | Motility, matrix degradation and epcam cleavage↑(34) |
|  |  | Ovarian cancer | ZDHHC12→claudin-3 palmitoylation↑→ accurate plasma membrane localization and protein stability↑ | Tumorigenic promotion effect↑(35) |
|  |  | Ovarian cancer | Claudin-4 or -7 palmitoylation→ EpCAM/claudins/CD82 complex↑ | Progression and metastasis↑(36) |

ADAM, a disintegrin and metalloproteinase; AR, androgen receptor; CRC, colorectal cancer; CSC, cancer stem cell; DNMT, DNA methyltransferases; DOCK, dedicator of cytokinesis; EGF, epithelial growth factor; EMT, epithelial-mesenchymal transition; EpCAM, epithelial cell adhesion molecule; EphA, ephrin type-A receptors; ERK, extracellular signal-regulated kinase; EZH, enhancer of zeste homolog; FAK, focal adhesion kinase; GEM, glycolipid-enriched membrane domain; GPR, G-protein-coupled receptor; HCC, hepatocellular carcinoma; hGH, human growth hormone; IL, interleukin; iPLA, calcium-independent phospholipase A; JAK, Janus kinase; LncRNA, long noncoding RNA; LUSC, lung squamous cell carcinoma; MAPK, mitogen-activated protein kinase; miR, microRNA; MMP, metalloproteinase; NSAIDs, non-steroidal anti-inflammatory drugs; NF-κB, nuclear factor-kappa B; PCNA, proliferating cell nuclear antigen; PDK, pyruvate dehydrogenase kinase; PGE, prostaglandin; PKC, protein kinase C; PPAR, peroxisome proliferator-activated receptor; PTM, post-translation modification; RCC, renal cell carcinoma; RhoGAP, Rho GTPase activating protein; RRP1B, ribosomal RNA processing 1B; RUNX, runt-related transcription factor; SCC, squamous cell carcinoma; STAT, signal transducer and activator of transcription; TACE, TNF-α converting enzyme; TF, transcription factor; TGF-β, transforming growth factor-β; TJ, tight junction; YAP, yes-associated protein; ZEB, zinc finger E-box binding homeobox; ZIP, Zrt-/Irt-like protein.

**References**

1. Chiang SK, Chang WC, Chen SE, Chang LC. DOCK1 regulates growth and motility through the RRP1B-claudin-1 pathway in claudin-low breast cancer cells. *Cancers (Basel)*. (2019) 11:1762. doi: 10.3390/cancers11111762

2. Lu Y, Wang L, Li H, Li Y, Ruan Y, Lin D, et al. SMAD2 inactivation inhibits CLDN6 methylation to suppress migration and invasion of breast cancer cells. *Int J Mol Sci*. (2017) 18:1863. doi: 10.3390/ijms18091863

3. Li CP, Cai MY, Jiang LJ, Mai SJ, Chen JW, Wang FW, et al. CLDN14 is epigenetically silenced by EZH2-mediated H3K27ME3 and is a novel prognostic biomarker in hepatocellular carcinoma. *Carcinogenesis*. (2016) 37:557-66. doi: 10.1093/carcin/bgw036

4. Du W, Xu X, Niu Q, Zhang X, Wei Y, Wang Z, et al. Spi-B-mediated silencing of claudin-2 promotes early dissemination of lung cancer cells from primary tumors. *Cancer Res*. (2017) 77:4809-22. doi: 10.1158/0008-5472.CAN-17-0020

5. Mattern J, Roghi CS, Hurtz M, Knäuper V, Edwards DR, Poghosyan Z. ADAM15 mediates upregulation of claudin-1 expression in breast cancer cells. *Sci Rep*. (2019) 9:12540. doi: 10.1038/s41598-019-49021-3

6. Akizuki R, Maruhashi R, Eguchi H, Kitabatake K, Tsukimoto M, Furuta T, et al. Decrease in paracellular permeability and chemosensitivity to doxorubicin by claudin-1 in spheroid culture models of human lung adenocarcinoma A549 cells. *Biochim Biophys Acta Mol Cell Res*. (2018) 1865:769-80. doi: 10.1016/j.bbamcr.2018.03.001

7. Maruhashi R, Eguchi H, Akizuki R, Hamada S, Furuta T, Matsunaga T, et al. Chrysin enhances anticancer drug-induced toxicity mediated by the reduction of claudin-1 and 11 expression in a spheroid culture model of lung squamous cell carcinoma cells. *Sci Rep*. (2019) 9:13753. doi: 10.1038/s41598-019-50276-z

8. Akimoto T, Takasawa A, Takasawa K, Aoyama T, Murata M, Osanai M, et al. Estrogen/GPR30 signaling contributes to the malignant potentials of ER-negative cervical adenocarcinoma via regulation of claudin-1 expression. *Neoplasia*. (2018) 20:1083-93. doi: 10.1016/j.neo.2018.08.010

9. Kyuno D, Kojima T, Yamaguchi H, Ito T, Kimura Y, Imamura M, et al. Protein kinase Cα inhibitor protects against downregulation of claudin-1 during epithelial-mesenchymal transition of pancreatic cancer. *Carcinogenesis*. (2013) 34:1232-43. doi: 10.1093/carcin/bgt057

10. Kim WK, Kwon Y, Jang M, Park M, Kim J, Cho S, et al. β-catenin activation down-regulates cell-cell junction-related genes and induces epithelial-to-mesenchymal transition in colorectal cancers. *Sci Rep*. (2019) 9:18440. doi: 10.1038/s41598-019-54890-9

11. Roy A, Ansari SA, Das K, Prasad R, Bhattacharya A, Mallik S, et al. Coagulation factor VIIa-mediated protease-activated receptor 2 activation leads to β-catenin accumulation via the AKT/GSK3β pathway and contributes to breast cancer progression. *J Biol Chem*. (2017) 292:13688-701. doi: 10.1074/jbc.M116.764670

12. de Souza WF, Fortunato-Miranda N, Robbs BK, de Araujo WM, de-Freitas-Junior JC, Bastos LG, et al. Claudin-3 overexpression increases the malignant potential of colorectal cancer cells: roles of ERK1/2 and PI3K-Akt as modulators of EGFR signaling. *PLoS One*. (2013) 8:e74994. doi: 10.1371/journal.pone.0074994

13. Rachakonda G, Vu T, Jin L, Samanta D, Datta PK. Role of TGF-β-induced Claudin-4 expression through c-Jun signaling in non-small cell lung cancer. *Cell Signal*. (2016) 28:1537-44. doi: 10.1016/j.cellsig.2016.07.006

14. Chen YJ, You ML, Chong QY, Pandey V, Zhuang QS, Liu DX, et al. Autocrine human growth hormone promotes invasive and cancer stem cell-like behavior of hepatocellular carcinoma cells by STAT3 dependent inhibition of claudin-1 expression. *Int J Mol Sci*. (2017) 18:1274. doi: 10.3390/ijms18061274

15. Li CF, Chen JY, Ho YH, Hsu WH, Wu LC, Lan HY, et al. Snail-induced claudin-11 prompts collective migration for tumour progression. *Nat Cell Biol*. (2019) 21:251-62. doi: 10.1038/s41556-018-0268-z

16. Liu M, Yang J, Zhang Y, Zhou Z, Cui X, Zhang L, et al. ZIP4 promotes pancreatic cancer progression by repressing ZO-1 and claudin-1 through a ZEB1-dependent transcriptional mechanism. *Clin Cancer Res*. (2018) 24:3186-96. doi: 10.1158/1078-0432.CCR-18-0263

17. Chang TL, Ito K, Ko TK, Liu Q, Salto-Tellez M, Yeoh KG, et al. Claudin-1 has tumor suppressive activity and is a direct target of RUNX3 in gastric epithelial cells. *Gastroenterology*. (2010) 138:255-65.e1-3. doi: 10.1053/j.gastro.2009.08.044

18. Ashikari D, Takayama KI, Obinata D, Takahashi S, Inoue S. CLDN8, an androgen-regulated gene, promotes prostate cancer cell proliferation and migration. *Cancer Sci*. (2017) 108:1386-93. doi: 10.1111/cas.13269

19. Mima S, Tsutsumi S, Ushijima H, Takeda M, Fukuda I, Yokomizo K, et al. Induction of claudin-4 by nonsteroidal anti-inflammatory drugs and its contribution to their chemopreventive effect. *Cancer Res*. (2005) 65:1868-76. doi: 10.1158/0008-5472.CAN-04-2770

20. Iitaka D, Moodley S, Shimizu H, Bai XH, Liu M. PKCδ-iPLA2-PGE2-PPARγ signaling cascade mediates TNF-α induced Claudin 1 expression in human lung carcinoma cells. *Cell Signal*. (2015) 27:568-77. doi: 10.1016/j.cellsig.2014.12.015

21. Zheng YF, Luo J, Gan GL, Li W. Overexpression of microRNA-98 inhibits cell proliferation and promotes cell apoptosis via claudin-1 in human colorectal carcinoma. *J Cell Biochem*. (2019) 120:6090-105. doi: 10.1002/jcb.27895

22. Zhang B, Lin Y, Bao Q, Zheng Y, Lan L. MiR-1193 inhibits the malignancy of cervical cancer cells by targeting claudin 7 (CLDN7). *Onco Targets Ther*. (2020) 13:4349-58. doi: 10.2147/OTT.S247115

23. Liu B, Lu B, Wang X, Jiang H, Kuang W. MiR-361-5p inhibits cell proliferation and induces cell apoptosis in retinoblastoma by negatively regulating CLDN8. *Childs Nerv Syst*. (2019) 35:1303-11. doi: 10.1007/s00381-019-04199-9

24. Sun H, Cui C, Xiao F, Wang H, Xu J, Shi X, et al. miR-486 regulates metastasis and chemosensitivity in hepatocellular carcinoma by targeting CLDN10 and CITRON. *Hepatol Res*. (2015) 45:1312-22. doi: 10.1111/hepr.12500

25. Yang P, Zhang M, Liu X, Pu H. MicroRNA-421 promotes the proliferation and metastasis of gastric cancer cells by targeting claudin-11. *Exp Ther Med*. (2017) 14:2625-32. doi: 10.3892/etm.2017.4798

26. Sun X, Cui S, Fu X, Liu C, Wang Z, Liu Y. MicroRNA-146-5p promotes proliferation, migration and invasion in lung cancer cells by targeting claudin-12. *Cancer Biomark*. (2019) 25:89-99. doi: 10.3233/CBM-182374

27. Zhang SJ, Feng JF, Wang L, Guo W, Du YW, Ming L, et al. miR-1303 targets claudin-18 gene to modulate proliferation and invasion of gastric cancer cells. *Dig Dis Sci*. (2014) 59:1754-63. doi: 10.1007/s10620-014-3107-5

28. Cheng B, Rong A, Zhou Q, Li W. LncRNA LINC00662 promotes colon cancer tumor growth and metastasis by competitively binding with miR-340-5p to regulate CLDN8/IL22 co-expression and activating ERK signaling pathway. *J Exp Clin Cancer Res*. (2020) 39:5. doi: 10.1186/s13046-019-1510-7

29. Zhang XZ, Mao HL, Zhang SJ, Sun L, Zhang WJ, Chen QZ, et al. lncRNA PCAT18 inhibits proliferation, migration and invasion of gastric cancer cells through miR-135b suppression to promote CLDN11 expression. *Life Sci*. (2020) 249:117478. doi: 10.1016/j.lfs.2020.117478

30. Owari T, Sasaki T, Fujii K, Fujiwara-Tani R, Kishi S, Mori S, et al. Role of nuclear claudin-4 in renal cell carcinoma. *Int J Mol Sci*. (2020) 21. doi: 10.3390/ijms21218340

31. French AD, Fiori JL, Camilli TC, Leotlela PD, O'Connell MP, et al. PKC and PKA phosphorylation affect the subcellular localization of claudin-1 in melanoma cells. *Int J Med Sci*. (2009) 6:93-101. doi: 10.7150/ijms.6.93

32. D'Souza T, Indig FE, Morin PJ. Phosphorylation of claudin-4 by PKCepsilon regulates tight junction barrier function in ovarian cancer cells. *Exp Cell Res*. (2007) 313:3364-75. doi: 10.1016/j.yexcr.2007.06.026

33. D'Souza T, Agarwal R, Morin PJ. Phosphorylation of claudin-3 at threonine 192 by cAMP-dependent protein kinase regulates tight junction barrier function in ovarian cancer cells. *J Biol Chem*. (2005) 280:26233-40. doi: 10.1074/jbc.M502003200

34. Heiler S, Mu W, Zöller M, Thuma F. The importance of claudin-7 palmitoylation on membrane subdomain localization and metastasis-promoting activities. *Cell Commun Signal*. (2015) 13:29. doi: 10.1186/s12964-015-0105-y

35. Yuan M, Chen X, Sun Y, Jiang L, Xia Z, Ye K, et al. ZDHHC12-mediated claudin-3 S-palmitoylation determines ovarian cancer progression. *Acta Pharm Sin B*. (2020) 10:1426-39. doi: 10.1016/j.apsb.2020.03.008

36. Tavsan Z, Ayar Kayalı H. EpCAM-claudin-tetraspanin-modulated ovarian cancer progression and drug resistance. *Cell Adh Migr*. (2020) 14:57-68. doi: 10.1080/19336918.2020.1732761

**Supplementary Table S2. Claudins activate or inhibit various signaling pathways in cancer with a context-dependent manner.**

| Signaling pathway | cancer type | Mechanism | Function |
| --- | --- | --- | --- |
| PI3K/Akt | CRC | Claudin-1→PI3K/Akt↑→ZEB-1↑→E-cadherin↓ | Invasion↑;anoikis↓(1) |
|  | EC | Claudin-6→PI3K/Akt/mTOR↑ | Proliferation, invasion and migration↑(2) |
|  | Osteosarcoma | Claudin-12→PI3K/Akt↑ | Proliferation and migration↑(3) |
|  | CRC | Claudin-1→Notch↑→HES1↑→PTEN↓→PI3K/Akt↑→β-catenin, protumorigenic M2 macrophages, and proinflammatory signals↑→epithelial homeostasis↓ | Mucosal inflammation, susceptibility to CAC and its malignancy↑(4) |
|  | LUAD | Claudin-18→PI3K/PDK1/Akt↓ | Proliferation and motility↓; anoikis↑(5) |
|  | Breast cancer | Claudin-6→SFKs↑→PI3K/Akt↑→RARγ and ERα↑ | Nuclear receptor activity↑(6) |
| Wnt/β-catenin | CRC | Claudin-1↑ or nuclear localization→E-cadherin↓; β-catenin/TCF↑ | EMT, growth of xenografted tumors and metastasis↑(7) |
|  | CRC | Claudin-3↓ and IL6/gp130/Stat3→Wnt/β-catenin↑ | Colon cancer development↑(8) |
|  | SACC | Claudin-7↓→Wnt/β-catenin↑ | Proliferation and metastasis↑(9) |
|  | Ovarian cancers | Claudin-3 and Claudin-4→E-cadherin↑→β-catenin↓ | Growth and metastasis↓(10) |
|  | LUSC | Claudin-3→Wnt/β-catenin↓ | EMT↓(11) |
| Hippo | Lung cancer | Claudin-18↓→p-LATS1/2↓→YAP↑ | Progenitor cell proliferation↑(12) |
|  | Lung cancer | Claudin-18.1↓→YAP/TAZ↑→IGF-1R and Akt phosphorylation↑ | Growth, proliferation, migration, invasion and anchorage-independent colony formation↑(13) |
|  | RCC | Nuclear claudin-4→nuclear translocation of YAP bound to claudin-4↑ | EMT↑(14) |
|  | OSCC | YAP1/ claudin-4 /ZO-2 complex→LATS1↓→nuclear translocation of YAP↑ | EMT, stemness, proliferation and invasion↑(15) |
|  | CRC | Claudin-2→YAP↑→miR-222-3p↑ | CSC renewal↑(16) |
| MAPK | HCC | Claudin-1→c-Abl-Ras-Raf-1-ERK1/2↑→Slug and ZEB1↑→E-cadherin↓; N-cadherin and Vimentin↑ | Adhesion↓; motility↑(17) |
|  | CRC | TNF-α→claudin-1↑→p-ERK and p-Src↑ | EMT, proliferation and migration↑(18) |
|  | Gastric cancer | Claudin-6→JNK↓ | Differentiation↑(19) |
|  | Breast cancer | Claudin-6→ASK1-p38/JNK↑→Bcl-2/Bax ratio↓ and caspase-3 cleavage↑ | Apoptosis↑(20) |
|  | CRC | Claudin-7↓→Rab25↓→p-Src and MAPK/ERK 1/2↑ | Proliferation, invasion and EMT↑(21) |
|  | Lung cancer | Claudin-7→MEK/ERK↓→death receptor 5↓ | TRAIL induced apoptosis↓(22) |
|  | Lung cancer | Claudin-7↓→MAPK/ERK↑ | Migration and invasion↑(23) |
| STAT | HCC | Claudin-9→TYK2↑→STAT3↑ | Metastatic ability↑(24) |
|  | Osteosarcoma | Claudin-10→JAK1/STAT1↑ | Metastatic ability↑(25) |
|  | LUSC | Claudin-12→TYK2/STAT1↑ | EMT↑(26) |
|  | HCC | Claudin-17→TYK2↑→STAT3↑ | Migration↑(27) |
| MMP | Melanoma | Claudin-1→MMP-2↑ | Invasion and motility↑(28) |
|  | OSCC | Claudin-1→MT1-MMP and MMP-2↑→cleavage of laminin-5 gamma2↑ | Invasion↑(29) |
|  | Gastric cancer | Claudin-6→claudin-1 membrane expression↑→MMP-2↑ | Migration and invasiveness↑(30) |
|  | HCC | Claudin-10→MMP2 and MT1-MMP↑ | Survival, motility and invasiveness↑(31) |
|  | Gastric cancer | Claudin-6→claudin-1↑→MMP-2↑ | Migration and invasiveness↑(30) |
|  | CRC | Claudin-8→p-ERK/Akt and MMP9↑ | Proliferation, migration and invasion↑(32) |
|  | Lung cancer | Claudin-18→ZO-2↑→MMP2↓ | Migration↓(33) |
|  | Gastric cancer | Claudin-4→MMP-2 and MMP-9↑ | Invasion and migration↑(34) |
| miRs | BLBC | Claudin 1→miRs (miR-9-5p, miR-9-3p, let-7c, miR-127-3p, miR-99a-5p, miR-129-5p, and miR-146a-5p) ↓ | Tumor progression↑(35) |
| Integrin | Breast cancer | Claudin-2/α(2)β(1)- and α(5)β(1)-integrin complexes↑→adhesion to fibronectin and type IV collagen↑ | Liver metastasis↑(36) |
|  | Lung cancer | Claudin-7/integrin β1 complexes→phospho-FAK↑ | Growth and cell cycle progression↓; cell-matrix interactions↑(37) |
|  | CRC | Claudin-7/ integrinβ1↓ | Viability, migration↑; apoptosis↓(38) |
|  | Gastric cancer | Claudin-4/ integrin β1→FAK-c-SRC↑→stem cell-associated proteins↑ | Chemotherapeutic sensitivity↓(39) |
|  | Lung cancer | Claudin-7/ integrinβ1↑ | Adhesion and motility↑(40) |
| EpCAM | Pancreatic cancer | EpCAM/claudin-7/tetraspanin-α6β4 complex in GEM→phosphorylated ezrin, cytoskeleton↑; EpCAM cleavage↑; MAPK and JNK↑ | Cell-cell adhesion↓; migration, proliferation and survival↑(41) |
|  | RCC | Claudin-7/EpCAM complex in TEM→pERK1/2↑, actin bundles association↑ | Proliferation, antiapoptotic proteins, drug resistance and motility↑(42) |
|  | CRC | EpCAM/claudin-7/CO-029/CD44v6 complex in TEM | Apoptosis resistance and metastasis↑(43) |
|  | Ovarian cancer | Claudin-4 or -7 palmitoylation→EpCAM-claudins-CD82 complex | Progression and metastasis↑(44) |
|  | CRC | Claudin-7/EpCAM in GEM→EpCAM cleaved by claudin-7-associated TACE and presenilin2↑→intracellular domain, EpIC↑ | EMT-TFs, fibronectin and vimentin↑(45) |

ASK, apoptosis signal‑regulating kinase; BLBC, basal-like breast cancer; CAC, colitis-associated cancer; CRC, colorectal cancer; CSC, cancer stem cell; EC, endometrial carcinoma; EMT, epithelial-mesenchymal transition; EpCAM, epithelial cell adhesion molecule; ER, estrogen receptor; ERK, extracellular signal-regulated kinase; FAK, focal adhesion kinase; GEM, glycolipid-enriched membrane domain; HCC, hepatocellular carcinoma; HES, hairy and enhancer of split; IGF-1R, insulin-like growth factor receptor-1; JAK, Janus kinase; JNK, c‑Jun N‑terminal kinase; LATS, large tumour suppressor; LUAD, lung adenocarcinoma; LUSC, lung squamous cell carcinoma; MAPK, mitogen-activated protein kinase; MEK, mitogen-activated extracellular signal-regulated kinase; miR, microRNA; MMP, metalloproteinase; MT1-MMP, membrane type 1-MMP; OSCC, oral squamous cell carcinoma; PDK, pyruvate dehydrogenase kinase; PTEN, phosphatase and tensin homologue; RAR, retinoic acid receptor; RCC, renal cell carcinoma; SACC, salivary adenoid cystic carcinoma; SFK, Src family kinases; STAT, signal transducer and activator of transcription; TACE, TNF-α converting enzyme; TAZ, transcriptional coactivator with PDZ-binding motif; TCF, T-cell factor; TEM, tetraspanin-enriched membrane microdomains; TF, transcription factor; TNF, umor necrosis factor; TRAIL, TNF-related apoptosis-inducing ligand; TYK, tyrosine kinase; YAP, yes-associated protein; ZEB, zinc finger E-box binding homeobox; ZO, zonula occludens.

**References**

1. Singh AB, Sharma A, Smith JJ, Krishnan M, Chen X, Eschrich S, et al. Claudin-1 up-regulates the repressor ZEB-1 to inhibit E-cadherin expression in colon cancer cells. *Gastroenterology*. (2011) 141:2140-53. doi: 10.1053/j.gastro.2011.08.038

2. Cao X, He GZ. Knockdown of CLDN6 inhibits cell proliferation and migration via PI3K/AKT/mTOR signaling pathway in endometrial carcinoma cell line HEC-1-B. *Onco Targets Ther*. (2018) 11:6351-60. doi: 10.2147/OTT.S174618

3. Tian X, He Y, Han Z, Su H, Chu C. The cytoplasmic expression of CLDN12 predicts an unfavorable prognosis and promotes proliferation and migration of osteosarcoma. *Cancer Manag Res*. (2019) 11:9339-51. doi: 10.2147/CMAR.S229441

4. Gowrikumar S, Ahmad R, Uppada SB, Washington MK, Shi C, Singh AB, et al. Upregulated claudin-1 expression promotes colitis-associated cancer by promoting β-catenin phosphorylation and activation in Notch/p-AKT-dependent manner. *Oncogene*. (2019) 38:5321-37. doi: 10.1038/s41388-019-0795-5

5. Shimobaba S, Taga S, Akizuki R, Hichino A, Endo S, Matsunaga T, et al. Claudin-18 inhibits cell proliferation and motility mediated by inhibition of phosphorylation of PDK1 and Akt in human lung adenocarcinoma A549 cells. *Biochim Biophys Acta*. (2016) 1863:1170-8. doi: 10.1016/j.bbamcr.2016.02.015

6. Sugimoto K, Ichikawa-Tomikawa N, Kashiwagi K, Endo C, Tanaka S, Sawada N, et al. Cell adhesion signals regulate the nuclear receptor activity. *Proc Natl Acad Sci U S A*. (2019) 116:24600-9. doi: 10.1073/pnas.1913346116

7. Dhawan P, Singh AB, Deane NG, No Y, Shiou SR, Schmidt C, et al. Claudin-1 regulates cellular transformation and metastatic behavior in colon cancer. *J Clin Invest*. (2005) 115:1765-76. doi: 10.1172/JCI24543

8. Ahmad R, Kumar B, Chen Z, Chen X, Müller D, Lele SM, et al. Loss of claudin-3 expression induces IL6/gp130/Stat3 signaling to promote colon cancer malignancy by hyperactivating Wnt/β-catenin signaling. *Oncogene*. (2017) 36:6592-604. doi: 10.1038/onc.2017.259

9. Ji H, Ding X, Zhang W, Zheng Y, Du H, Zheng Y, et al. Claudin-7 inhibits proliferation and metastasis in salivary adenoid cystic carcinoma through Wnt/β-Catenin signaling. *Cell Transplant*. (2020) 29:963689720943583. doi: 10.1177/0963689720943583

10. Shang X, Lin X, Alvarez E, Manorek G, Howell SB. Tight junction proteins claudin-3 and claudin-4 control tumor growth and metastases. *Neoplasia*. (2012) 14:974-85. doi: 10.1593/neo.12942

11. Che J, Yue D, Zhang B, Zhang H, Huo Y, Gao L, et al. Claudin-3 inhibits lung squamous cell carcinoma cell epithelial-mesenchymal transition and invasion via suppression of the Wnt/β-catenin signaling pathway. *Int J Med Sci*. (2018) 15:339-51. doi: 10.7150/ijms.22927

12. Zhou B, Flodby P, Luo J, Castillo DR, Liu Y, Yu FX, et al. Claudin-18-mediated YAP activity regulates lung stem and progenitor cell homeostasis and tumorigenesis. *J Clin Invest*. (2018) 128:970-84. doi: 10.1172/JCI90429

13. Luo J, Chimge NO, Zhou B, Flodby P, Castaldi A, Firth AL, et al. CLDN18.1 attenuates malignancy and related signaling pathways of lung adenocarcinoma in vivo and in vitro. *Int J Cancer*. (2018) 143:3169-80. doi: 10.1002/ijc.31734

14. Owari T, Sasaki T, Fujii K, Fujiwara-Tani R, Kishi S, Mori S, et al. Role of nuclear claudin-4 in renal cell carcinoma. *Int J Mol Sci*. (2020) 21. doi: 10.3390/ijms21218340

15. Nakashima C, Yamamoto K, Kishi S, Sasaki T, Ohmori H, Fujiwara-Tani R, et al. Clostridium perfringens enterotoxin induces claudin-4 to activate YAP in oral squamous cell carcinomas. *Oncotarget*. (2020) 11:309-21. doi: 10.18632/oncotarget.27424

16. Paquet-Fifield S, Koh SL, Cheng L, Beyit LM, Shembrey C, Mølck C, et al. Tight junction protein claudin-2 promotes self-renewal of human colorectal cancer stem-like cells. *Cancer Res*. (2018) 78:2925-38. doi: 10.1158/0008-5472.CAN-17-1869

17. Suh Y, Yoon CH, Kim RK, Lim EJ, Oh YS, Hwang SG, et al. Claudin-1 induces epithelial-mesenchymal transition through activation of the c-Abl-ERK signaling pathway in human liver cells. *Oncogene*. (2013) 32:4873-82. doi: 10.1038/onc.2012.505

18. Bhat AA, Ahmad R, Uppada SB, Singh AB, Dhawan P. Claudin-1 promotes TNF-α-induced epithelial-mesenchymal transition and migration in colorectal adenocarcinoma cells. *Exp Cell Res*. (2016) 349:119-27. doi: 10.1016/j.yexcr.2016.10.005

19. Lu YZ, Li Y, Zhang T, Han ST. Claudin-6 is down-regulated in gastric cancer and its potential pathway. *Cancer Biomark*. (2020) 28:329-40. doi: 10.3233/CBM-201554

20. Guo Y, Lin D, Zhang M, Zhang X, Li Y, Yang R, et al. CLDN6-induced apoptosis via regulating ASK1-p38/JNK signaling in breast cancer MCF-7 cells. *Int J Oncol*. (2016) 48:2435-44. doi: 10.3892/ijo.2016.3469

21. Bhat AA, Pope JL, Smith JJ, Ahmad R, Chen X, Washington MK, et al. Claudin-7 expression induces mesenchymal to epithelial transformation (MET) to inhibit colon tumorigenesis. *Oncogene*. (2015) 34:4570-80. doi: 10.1038/onc.2014.385

22. Xia P, Wang W, Bai Y. Claudin-7 suppresses the cytotoxicity of TRAIL-expressing mesenchymal stem cells in H460 human non-small cell lung cancer cells. *Apoptosis*. (2014) 19:491-505. doi: 10.1007/s10495-013-0938-z

23. Lu Z, Ding L, Hong H, Hoggard J, Lu Q, Chen YH. Claudin-7 inhibits human lung cancer cell migration and invasion through ERK/MAPK signaling pathway. *Exp Cell Res*. (2011) 317:1935-46. doi: 10.1016/j.yexcr.2011.05.019

24. Liu H, Wang M, Liang N, Guan L. Claudin-9 enhances the metastatic potential of hepatocytes via Tyk2/Stat3 signaling. *Turk J Gastroenterol*. (2019) 30:722-31. doi: 10.5152/tjg.2019.18513

25. Zhang X, Wang X, Wang A, Li Q, Zhou M, Li T. CLDN10 promotes a malignant phenotype of osteosarcoma cells via JAK1/Stat1 signaling. *J Cell Commun Signal*. (2019) 13:395-405. doi: 10.1007/s12079-019-00509-7

26. Sun L, Feng L, Cui J. Increased expression of claudin-12 promotes the metastatic phenotype of human bronchial epithelial cells and is associated with poor prognosis in lung squamous cell carcinoma. *Exp Ther Med*. (2019) 17:165-74. doi: 10.3892/etm.2018.6964

27. Sun L, Feng L, Cui J. Increased expression of claudin-17 promotes a malignant phenotype in hepatocyte via Tyk2/Stat3 signaling and is associated with poor prognosis in patients with hepatocellular carcinoma. *Diagn Pathol*. (2018) 13:72. doi: 10.1186/s13000-018-0749-1

28. Leotlela PD, Wade MS, Duray PH, Rhode MJ, Brown HF, Rosenthal DT, et al. Claudin-1 overexpression in melanoma is regulated by PKC and contributes to melanoma cell motility. *Oncogene*. (2007) 26:3846-56. doi: 10.1038/sj.onc.1210155

29. Oku N, Sasabe E, Ueta E, Yamamoto T, Osaki T. Tight junction protein claudin-1 enhances the invasive activity of oral squamous cell carcinoma cells by promoting cleavage of laminin-5 gamma2 chain via matrix metalloproteinase (MMP)-2 and membrane-type MMP-1. *Cancer Res*. (2006) 66:5251-7. doi: 10.1158/0008-5472.CAN-05-4478

30. Torres-Martínez AC, Gallardo-Vera JF, Lara-Holguin AN, Montaño LF, Rendón-Huerta EP. Claudin-6 enhances cell invasiveness through claudin-1 in AGS human adenocarcinoma gastric cancer cells. *Exp Cell Res*. (2017) 350:226-35. doi: 10.1016/j.yexcr.2016.11.025

31. Ip YC, Cheung ST, Lee YT, Ho JC, Fan ST. Inhibition of hepatocellular carcinoma invasion by suppression of claudin-10 in HLE cells. *Mol Cancer Ther*. (2007) 6:2858-67. doi: 10.1158/1535-7163.MCT-07-0453

32. Cheng B, Rong A, Zhou Q, Li W. CLDN8 promotes colorectal cancer cell proliferation, migration, and invasion by activating MAPK/ERK signaling. *Cancer Manag Res*. (2019) 11:3741-51. doi: 10.2147/CMAR.S189558

33. Akizuki R, Eguchi H, Endo S, Matsunaga T, Ikari A. ZO-2 Suppresses Cell Migration Mediated by a Reduction in Matrix Metalloproteinase 2 in Claudin-18-Expressing Lung Adenocarcinoma A549 Cells. *Biol Pharm Bull*. (2019) 42:247-54. doi: 10.1248/bpb.b18-00670

34. Hwang TL, Changchien TT, Wang CC, Wu CM. Claudin-4 expression in gastric cancer cells enhances the invasion and is associated with the increased level of matrix metalloproteinase-2 and -9 expression. *Oncol Lett*. (2014) 8:1367-71. doi: 10.3892/ol.2014.2295

35. Majer A, Blanchard AA, Medina S, Booth SA, Myal Y. Claudin 1 expression levels affect miRNA dynamics in human basal-like breast cancer cells. *DNA Cell Biol*. (2016) 35:328-39. doi: 10.1089/dna.2015.3188

36. Tabariès S, Dong Z, Annis MG, Omeroglu A, Pepin F, Ouellet V, et al. Claudin-2 is selectively enriched in and promotes the formation of breast cancer liver metastases through engagement of integrin complexes. *Oncogene*. (2011) 30:1318-28. doi: 10.1038/onc.2010.518

37. Lu Z, Kim DH, Fan J, Lu Q, Verbanac K, Ding L, et al. A non-tight junction function of claudin-7-Interaction with integrin signaling in suppressing lung cancer cell proliferation and detachment. *Mol Cancer*. (2015) 14:120. doi: 10.1186/s12943-015-0387-0

38. Li W, Xu C, Wang K, Ding Y, Ding L. Non-tight junction-related function of claudin-7 in interacting with integrinβ1 to suppress colorectal cancer cell proliferation and migration. *Cancer Manag Res*. (2019) 11:1443-51. doi: 10.2147/CMAR.S188020

39. Nishiguchi Y, Fujiwara-Tani R, Sasaki T, Luo Y, Ohmori H, Kishi S, et al. Targeting claudin-4 enhances CDDP-chemosensitivity in gastric cancer. *Oncotarget*. (2019) 10:2189-202. doi: 10.18632/oncotarget.26758

40. Kim DH, Lu Q, Chen YH. Claudin-7 modulates cell-matrix adhesion that controls cell migration, invasion and attachment of human HCC827 lung cancer cells. *Oncol Lett*. (2019) 17:2890-6. doi: 10.3892/ol.2019.9909

41. Thuma F, Zöller M. EpCAM-associated claudin-7 supports lymphatic spread and drug resistance in rat pancreatic cancer. *Int J Cancer*. (2013) 133:855-66. doi: 10.1002/ijc.28085

42. Nübel T, Preobraschenski J, Tuncay H, Weiss T, Kuhn S, Ladwein M, et al. Claudin-7 regulates EpCAM-mediated functions in tumor progression. *Mol Cancer Res*. (2009) 7:285-99. doi: 10.1158/1541-7786.MCR-08-0200

43. Kuhn S, Koch M, Nübel T, Ladwein M, Antolovic D, Klingbeil P, et al. A complex of EpCAM, claudin-7, CD44 variant isoforms, and tetraspanins promotes colorectal cancer progression. *Mol Cancer Res*. (2007) 5:553-67. doi: 10.1158/1541-7786.MCR-06-0384

44. Tavsan Z, Ayar Kayalı H. EpCAM-claudin-tetraspanin-modulated ovarian cancer progression and drug resistance. *Cell Adh Migr*. (2020) 14:57-68. doi: 10.1080/19336918.2020.1732761

45. Philip R, Heiler S, Mu W, Büchler MW, Zöller M, Thuma F. Claudin-7 promotes the epithelial-mesenchymal transition in human colorectal cancer. *Oncotarget*. (2015) 6:2046-63. doi: 10.18632/oncotarget.2858

**Supplementary Table S3.The genetic alterations of claudin genes in cancer.**

| Claudin gene | Total frequency | The common cancers affected by genetic alterations of *CLDNs* and the frequency in individual cancer types |
| --- | --- | --- |
| *CLDN1* | 5.21% | Amplification: Lung squ (29.77%); Esophagus (17.58%); Ovarian (15.07%); Cervical (13.80%); Head & neck (13.38%)  Deep deletion: Prostate (1.42%)  Missense mutation: Uterine (3.02%); Melanoma (2.03%) |
| *CLDN2* | 0.96% | Amplification: Esophagus (1.65%); Lung squ (1.03%); ccRCC (1.17%)  Missense mutation: Uterine (1.89%); Melanoma (1.8%); Cervical (1.01%) |
| *CLDN3* | 1.04% | Amplification: Esophagus (2.2%); Stomach (2.05%); Ovarian (1.71%)  Missense mutation: Melanoma (0.9%) |
| *CLDN4* | 1.36% | Amplification: Stomach (2.05%); Ovarian (2.23%); Esophagus (2.2%)  Missense mutation: Melanoma (2.93%); Uterine (1.51%); Stomach (1.14%) |
| *CLDN5* | 1.70% | Amplification: Lung squ (3.29%); Sarcoma (3.92%); Bladder (2.68%); Ovarian (2.74%); Melanoma (2.48%)  Deep deletion: Esophagus (1.1%); Lung squ (0.82%)  Missense mutation: Stomach (1.14%); Uterine (1.13%); Liver (1.08%) |
| *CLDN6* | 1.35% | Amplification: Breast (3.69%); Ovarian (1.03%); Prostate (1.21%)  Deep deletion: Bladder (1.7%)  Missense mutation: Stomach (2.72%); Melanoma (2.25%) |
| *CLDN7* | 1.06% | Amplification: Ovarian (1.20%)  Deep deletion: Prostate (3.64%); Liver (2.42%); Colorectal (1.35%)  Missense mutation: Uterine (0.76%); Melanoma (0.68%); Colorectal (0.51%) |
| *CLDN8* | 0.80% | Amplification: Sarcoma (1.57%); Bladder (0.97%)  Deep deletion: Stomach (0.91%)  Missense mutation: Uterine (2.27%); Colorectal (1.68%); Lung adeno (1.24%) |
| *CLDN9* | 1.23% | Amplification: Breast (3.69%); Ovarian (1.03%); Prostate (1.21%)  Deep deletion: Bladder (1.70%)  Missense mutation: Stomach (2.72%); Uterine (1.13%); Lung squ (0.82%) |
| *CLDN10* | 1.84% | Amplification: Ovarian (12.57%); Liver (2.42%); Bladder (1.95%)  Deep deletion: Prostate (0.81%); Bladder (0.97%)  Missense mutation: Melanoma (2.93%); Uterine (3.97%); Colorectal (1.85%); Lung adeno (1.24%) |
| *CLDN11* | 6.25% | Amplification: Lung squ (35.73%); Ovarian (23.46%); Esophagus (19.78%); Cervical (15.49%); Head & neck (13.0%)  Missense mutation: Uterine (1.89%); Melanoma (1.35%) |
| *CLDN12* | 1.86% | Amplification: Esophagus (9.89%); Stomach (4.55%); Lung squ (3.9%)  Missense mutation: Lung squ (1.64%); Uterine (1.51%); Stomach (1.14%) |
| *CLDN14* | 1.05% | Amplification: Bladder (1.22%); Ovarian (1.2%); Cervical (1.01%)  Deep deletion: Stomach (1.14%)  Missense mutation: Melanoma (1.58%); Uterine (1.51%); Lung adeno (1.06%) |
| *CLDN15* | 1.78% | Amplification: Esophagus (8.24%); Lung squ (3.7%); Stomach (3.64%); Head & neck (3.44%)  Missense mutation: Uterine (2.46%); Melanoma (1.58%) |
| *CLDN16* | 5.55% | Amplification: Lung squ (29.16%); Esophagus (17.58%); Ovarian (15.07%); Cervical (14.14%); Head & neck (12.81%)  Deep deletion: Prostate (1.62%)  Missense mutation: Uterine (2.84%); Melanoma (2.48%); Lung adeno (1.41%) |
| *CLDN17* | 0.98% | Amplification: Sarcoma (1.57%)  Missense mutation: Uterine (3.21%); Melanoma (1.8%); Lung adeno (1.59%); Colorectal (1.35%)  Deep deletion: Stomach (0.91%) |
| *CLDN18* | 2.40% | Amplification: Lung squ (9.03%); Cervical (7.07%); Esophagus (6.59%); Head & neck (4.97%)  Missense mutation: Uterine (3.40%); Melanoma (2.7%); Colorectal (1.85%)  Fusion: Stomach (2.73%) |
| *CLDN19* | 1.48% | Amplification: Ovarian (6.51%); Bladder (3.89%); Uterine (2.65%)  Missense mutation: Uterine (2.08%); Melanoma (1.58%); Stomach (1.14%) |
| *CLDN20* | 1.00% | Amplification: Sarcoma (3.14%)  Deep deletion: Uveal melanoma (7.5%); Liver (1.61%); Ovarian (1.2%)  Missense mutation: Uterine (1.51%); Melanoma (0.9%); Stomach (0.91%) |
| *CLDN22* | 2.11% | Deep deletion: Lung squ (4.52%); Stomach (3.64%); Sarcoma (3.53%)  Missense mutation: Uterine (2.08%); Melanoma (1.13%); Stomach (0.91%) |
| *CLDN23* | 1.73% | Amplification: Stomach (3.86%); Esophagus (3.3%)  Deep deletion: Liver (7.53%); Bladder (6.57%); Ovarian (5.82%); Colorectal (5.39%)  Missense mutation: Stomach (0.91%); Uterine (0.76%) |
| *CLDN24* | 1.75% | Amplification: Esophagus (1.1%)  Deep deletion: Lung squ (4.52%); Stomach (3.64%); Esophagus (3.3%); Sarcoma (3.53%) |
| *CLDN25* | 1.46% | Deep deletion: Cervical (3.03%); Melanoma (2.93%); Stomach (1.14%)  Missense mutation: Uterine (3.02%); Stomach (1.36%); Lung squ (1.85%); Lung adeno (1.24%) |

Data was obtained from cBioPortal using the TCGA PanCancer Atlas studies. The total frequency includes the frequencies of amplification, deep deletion, truncating mutation, missense mutation and fusion. ccRCC, clear cell renal cell cancer; TCGA, the cancer genome atlas.

**Supplementary Table S4. Genetic studies of claudins using genetically engineered mouse models**

| Claudin | Tissue or organ | Phenotype |
| --- | --- | --- |
| Knockout (including global, tissue specific, inducibale) | | |
| 1 | Sebaceous glands | Incomplete holocrine secretion; Barrier leakage.(1) |
|  | Skin | Died within 1d of birth with wrinkled skin; Epidermal barrier disruption; Layered organization of keratinocytes appeared to be normal.(2) |
| 2 | Intestine | Limits progression of immune-mediated colitis; Insufficient fecal hydration; Increased net intestinal calcium absorption; Reduced paracellular calcium permeability in the colon.(3, 4) |
|  | Kidney | Reabsorption deficiency; Hypercalciuria and papillary nephrocalcinosis.(4) |
|  | Hepatobiliary system | Gallstones result from concentrated bile.(5) |
| 3 | Intestine | Dedifferentiated and leaky colonic epithelium; Invasive adenocarcinoma development.(6) |
|  | Hepatobiliary system | Cholesterol gallstone disease result from increased paracellular phosphate ion permeability.(7) |
|  | Testis | Prolonged preleptotene phase during spermatogenesis and delaying in spermatocyte migration across the BTB.(8) |
|  | Skin | Sweat leakage.(9) |
| 4 | Lung | Loss of protection against lung injury.(10, 11) |
|  | Urinary system | Hydronephrosis due to urothelial hyperplasia.(12) |
| 5 | Brain | The blood-brain barrier (BBB) against small molecules was selectively affected.(13) |
| 7 | Intestine | Died during the perinatal period with increased pFlux for small organic solutes, mucosal ulcerations, and colonic inflammation in conventional knockout mice; Dying state with inflammatory cell infiltration, atypical hyperplasia and adenoma in inducible conditional knockout mice.(14-17) |
| 10 | Kidney | Hypermagnesemia and nephrocalcinosis.(18) |
| 11 | Cochlea | Endocochlear potentials decreased and hearing thresholds elevated.(19) |
| 12 | Kidney | Calcium permeability was compensated by reduced claudin-14 expression.(20) |
| 14 | Cochlea | Deaf result from hair cell loss.(21, 22) |
| 15 | Intestine | Na+ permeability and glucose absorption dysregulation; Enlarged upper small intestinal result from proliferation of normal cryptic cells but without neoplasia.(23-25) |
| 16 | Tooth | Enamel phenotype closely resembling humanamelogenesis imperfecta (AI).(26) |
|  | Kidney | Transport defect for Mg(2+) and Ca(2+); Renal nephrocalcinosis.(27-29) |
| 18 | Stomach | No paracellular permeability defects; Up-regulation of claudin-2; Anion permeability alteration secondary to transcellular anion transporter expression/function.(30) |
|  | Stomach | Low levels of inflammation, increased cell proliferation, spasmolytic polypeptide-expressing metaplasia (SPEM) by 7 weeks after birth; Intraepithelial neoplasia invaded submucosa by 20 to 30 weeks; Dysplastic polypoid tumors with invasive glands invaded serosa by 2 years.(31) |
|  | Lung | Lung enlargement, increased proliferation of AT2 cells, lung adenocarcinomas (LuAd) development with age; Increased claudin 3 and claudin 4 expression, increased alveolar fluid clearance (AFC); Epithelial barrier dysfunction, injury, and impaired alveolarization.(32-34) |
| 18.2 | Stomach | Paracellular H(+) leak; Atrophic gastritis and SPEM; Gastric tumorigenesis.(35, 36) |
| 19 | Kidney | Chronic renal wasting of magnesium and calcium.(37) |
|  | Schwann cells | Behavioral abnormalities result from defective nerve conduction.(38) |
| Transgenic overexpression | | |
| 1 | Intestine | Mucosal inflammation; Increases susceptibility to colitis-associated cancer (CAC); Early onset of adenoma and tumorigenesis (concomitant with *APC* knockout).(39-41) |
|  | Kidney | Proteinuria.(42) |
| 2 | Intestine | Increased colonocyte proliferation and protection against colitis-induced colonocyte death.(43) |
| 6 | Skin | Die within 2 days of birth with a epidermal phenotype very reminiscent of that in pre-term infant skin.(44) |

**References**

1. Atsugi T, Yokouchi M, Hirano T, Hirabayashi A, Nagai T, Ohyama M, et al. Holocrine secretion occurs outside the tight junction barrier in multicellular glands: lessons from claudin-1-deficient mice. *J Invest Dermatol*. (2020) 140:298-308.e5. doi: 10.1016/j.jid.2019.06.150

2. Furuse M, Hata M, Furuse K, Yoshida Y, Haratake A, Sugitani Y, et al. Claudin-based tight junctions are crucial for the mammalian epidermal barrier: a lesson from claudin-1-deficient mice. *J Cell Biol*. (2002) 156:1099-111. doi: 10.1083/jcb.200110122

3. Raju P, Shashikanth N, Tsai PY, Pongkorpsakol P, Chanez-Paredes S, Steinhagen PR, et al. Inactivation of paracellular cation-selective claudin-2 channels attenuates immune-mediated experimental colitis in mice. *J Clin Invest*. (2020) 130:5197-208. doi: 10.1172/JCI138697

4. Curry JN, Saurette M, Askari M, Pei L, Filla MB, Beggs MR, et al. Claudin-2 deficiency associates with hypercalciuria in mice and human kidney stone disease. *J Clin Invest*. (2020) 130:1948-60. doi: 10.1172/JCI127750

5. Matsumoto K, Imasato M, Yamazaki Y, Tanaka H, Watanabe M, Eguchi H, et al. Claudin 2 deficiency reduces bile flow and increases susceptibility to cholesterol gallstone disease in mice. *Gastroenterology*. (2014) 147:1134-45.e10. doi: 10.1053/j.gastro.2014.07.033

6. Ahmad R, Kumar B, Chen Z, Chen X, Müller D, Lele SM, et al. Loss of claudin-3 expression induces IL6/gp130/Stat3 signaling to promote colon cancer malignancy by hyperactivating Wnt/β-catenin signaling. *Oncogene*. (2017) 36:6592-604. doi: 10.1038/onc.2017.259

7. Tanaka H, Imasato M, Yamazaki Y, Matsumoto K, Kunimoto K, Delpierre J, et al. Claudin-3 regulates bile canalicular paracellular barrier and cholesterol gallstone core formation in mice. *J Hepatol*. (2018) 69:1308-16. doi: 10.1016/j.jhep.2018.08.025

8. Chihara M, Ikebuchi R, Otsuka S, Ichii O, Hashimoto Y, Suzuki A, et al. Mice stage-specific claudin 3 expression regulates progression of meiosis in early stage spermatocytes. *Biol Reprod*. (2013) 89:3. doi: 10.1095/biolreprod.113.107847

9. Yamaga K, Murota H, Tamura A, Miyata H, Ohmi M, Kikuta J, et al. Claudin-3 loss causes leakage of sweat from the sweat gland to contribute to the pathogenesis of atopic dermatitis. *J Invest Dermatol*. (2018) 138:1279-87. doi: 10.1016/j.jid.2017.11.040

10. Kage H, Flodby P, Gao D, Kim YH, Marconett CN, DeMaio L, et al. Claudin 4 knockout mice: normal physiological phenotype with increased susceptibility to lung injury. *Am J Physiol Lung Cell Mol Physiol*. (2014) 307:L524-36. doi: 10.1152/ajplung.00077.2014

11. Wray C, Mao Y, Pan J, Chandrasena A, Piasta F, Frank JA. Claudin-4 augments alveolar epithelial barrier function and is induced in acute lung injury. *Am J Physiol Lung Cell Mol Physiol*. (2009) 297:L219-27. doi: 10.1152/ajplung.00043.2009

12. Fujita H, Hamazaki Y, Noda Y, Oshima M, Minato N. Claudin-4 deficiency results in urothelial hyperplasia and lethal hydronephrosis. *PLoS One*. (2012) 7:e52272. doi: 10.1371/journal.pone.0052272

13. Nitta T, Hata M, Gotoh S, Seo Y, Sasaki H, Hashimoto N, et al. Size-selective loosening of the blood-brain barrier in claudin-5-deficient mice. *J Cell Biol*. (2003) 161:653-60. doi: 10.1083/jcb.200302070

14. Tanaka H, Takechi M, Kiyonari H, Shioi G, Tamura A, Tsukita S. Intestinal deletion of Claudin-7 enhances paracellular organic solute flux and initiates colonic inflammation in mice. *Gut*. (2015) 64:1529-38. doi: 10.1136/gutjnl-2014-308419

15. Ding L, Lu Z, Foreman O, Tatum R, Lu Q, Renegar R, et al. Inflammation and disruption of the mucosal architecture in claudin-7-deficient mice. *Gastroenterology*. (2012) 142:305-15. doi: 10.1053/j.gastro.2011.10.025

16. Xu C, Wang K, Ding YH, Li WJ, Ding L. Claudin-7 gene knockout causes destruction of intestinal structure and animal death in mice. *World J Gastroenterol*. (2019) 25:584-99. doi: 10.3748/wjg.v25.i5.584

17. Li WJ, Xu C, Wang K, Li TY, Wang XN, Yang H, et al. Severe intestinal inflammation in the small intestine of mice induced by controllable deletion of claudin-7. *Dig Dis Sci*. (2018) 63:1200-9. doi: 10.1007/s10620-018-4973-z

18. Breiderhoff T, Himmerkus N, Stuiver M, Mutig K, Will C, Meij IC, et al. Deletion of claudin-10 (Cldn10) in the thick ascending limb impairs paracellular sodium permeability and leads to hypermagnesemia and nephrocalcinosis. *Proc Natl Acad Sci U S A*. (2012) 109:14241-6. doi: 10.1073/pnas.1203834109

19. Gow A, Davies C, Southwood CM, Frolenkov G, Chrustowski M, Ng L, et al. Deafness in Claudin 11-null mice reveals the critical contribution of basal cell tight junctions to stria vascularis function. *J Neurosci*. (2004) 24:7051-62. doi: 10.1523/JNEUROSCI.1640-04.2004

20. Plain A, Pan W, O'Neill D, Ure M, Beggs MR, et al. Claudin-12 knockout mice demonstrate reduced proximal tubule calcium permeability. *Int J Mol Sci*. (2020) 21:2074. doi: 10.3390/ijms21062074

21. Claußen M, Schulze J, Nothwang HG. Loss of inner hair cell ribbon synapses and auditory nerve fiber regression in Cldn14 knockout mice. *Hear Res*. (2020) 391:107950. doi: 10.1016/j.heares.2020.107950

22. Ben-Yosef T, Belyantseva IA, Saunders TL, Hughes ED, Kawamoto K, Van Itallie CM, et al. Claudin 14 knockout mice, a model for autosomal recessive deafness DFNB29, are deaf due to cochlear hair cell degeneration. *Hum Mol Genet*. (2003) 12:2049-61. doi: 10.1093/hmg/ddg210

23. Nakayama M, Ishizuka N, Hempstock W, Ikari A, Hayashi H. Na(+)-coupled nutrient cotransport induced luminal negative potential and claudin-15 play an important role in paracellular Na(+) recycling in mouse small intestine. *Int J Mol Sci*. (2020) 21:376. doi: 10.3390/ijms21020376

24. Tamura A, Hayashi H, Imasato M, Yamazaki Y, Hagiwara A, Wada M, et al. Loss of claudin-15, but not claudin-2, causes Na+ deficiency and glucose malabsorption in mouse small intestine. *Gastroenterology*. (2011) 140:913-23. doi: 10.1053/j.gastro.2010.08.006

25. Tamura A, Kitano Y, Hata M, Katsuno T, Moriwaki K, Sasaki H, et al. Megaintestine in claudin-15-deficient mice. *Gastroenterology*. (2008) 134:523-34. doi: 10.1053/j.gastro.2007.11.040

26. Bardet C, Courson F, Wu Y, Khaddam M, Salmon B, Ribes S, et al. Claudin-16 deficiency impairs tight junction function in ameloblasts, leading to abnormal enamel formation. *J Bone Miner Res*. (2016) 31:498-513. doi: 10.1002/jbmr.2726

27. Shan Q, Himmerkus N, Hou J, Goodenough DA, Bleich M. Insights into driving forces and paracellular permeability from claudin-16 knockdown mouse. *Ann N Y Acad Sci*. (2009) 1165:148-51. doi: 10.1111/j.1749-6632.2009.04041.x

28. Himmerkus N, Shan Q, Goerke B, Hou J, Goodenough DA, Bleich M. Salt and acid-base metabolism in claudin-16 knockdown mice: impact for the pathophysiology of FHHNC patients. *Am J Physiol Renal Physiol*. (2008) 295:F1641-7. doi: 10.1152/ajprenal.90388.2008

29. Hou J, Shan Q, Wang T, Gomes AS, Yan Q, Paul DL, et al. Transgenic RNAi depletion of claudin-16 and the renal handling of magnesium. *J Biol Chem*. (2007) 282:17114-22. doi: 10.1074/jbc.M700632200

30. Caron TJ, Scott KE, Sinha N, Muthupalani S, Baqai M, Ang LH, et al. Claudin-18 loss alters transcellular chloride flux but not tight junction ion selectivity in gastric epithelial cells. *Cell Mol Gastroenterol Hepatol*. (2020) :S2352-45X(20)30168-5. doi: 10.1016/j.jcmgh.2020.10.005

31. Hagen SJ, Ang LH, Zheng Y, Karahan SN, Wu J, Wang YE, et al. Loss of tight junction protein claudin 18 promotes progressive neoplasia development in mouse stomach. *Gastroenterology*. (2018) 155:1852-67. doi: 10.1053/j.gastro.2018.08.041

32. Zhou B, Flodby P, Luo J, Castillo DR, Liu Y, Yu FX, et al. Claudin-18-mediated YAP activity regulates lung stem and progenitor cell homeostasis and tumorigenesis. *J Clin Invest*. (2018) 128:970-84. doi: 10.1172/JCI90429

33. Li G, Flodby P, Luo J, Kage H, Sipos A, Gao D, et al. Knockout mice reveal key roles for claudin 18 in alveolar barrier properties and fluid homeostasis. *Am J Respir Cell Mol Biol*. (2014) 51:210-22. doi: 10.1165/rcmb.2013-0353OC

34. LaFemina MJ, Sutherland KM, Bentley T, Gonzales LW, Allen L, Chapin CJ, et al. Claudin-18 deficiency results in alveolar barrier dysfunction and impaired alveologenesis in mice. *Am J Respir Cell Mol Biol*. (2014) 51:550-8. doi: 10.1165/rcmb.2013-0456OC

35. Hayashi D, Tamura A, Tanaka H, Yamazaki Y, Watanabe S, Suzuki K, et al. Deficiency of claudin-18 causes paracellular H+ leakage, up-regulation of interleukin-1β, and atrophic gastritis in mice. *Gastroenterology*. (2012) 142:292-304. doi: 10.1053/j.gastro.2011.10.040

36. Suzuki K, Sentani K, Tanaka H, Yano T, Suzuki K, Oshima M, et al. Deficiency of stomach-type claudin-18 in mice induces gastric tumor formation independent of H pylori infection. *Cell Mol Gastroenterol Hepatol*. (2019) 8:119-42. doi: 10.1016/j.jcmgh.2019.03.003

37. Hou J, Renigunta A, Gomes AS, Hou M, Paul DL, Waldegger S, et al. Claudin-16 and claudin-19 interaction is required for their assembly into tight junctions and for renal reabsorption of magnesium. *Proc Natl Acad Sci U S A*. (2009) 106:15350-5. doi: 10.1073/pnas.0907724106

38. Miyamoto T, Morita K, Takemoto D, Takeuchi K, Kitano Y, Miyakawa T, et al. Tight junctions in Schwann cells of peripheral myelinated axons: a lesson from claudin-19-deficient mice. *J Cell Biol*. (2005) 169:527-38. doi: 10.1083/jcb.200501154

39. Gowrikumar S, Ahmad R, Uppada SB, Washington MK, Shi C, Singh AB, et al. Upregulated claudin-1 expression promotes colitis-associated cancer by promoting β-catenin phosphorylation and activation in Notch/p-AKT-dependent manner. *Oncogene*. (2019) 38:5321-37. doi: 10.1038/s41388-019-0795-5

40. Pope JL, Bhat AA, Sharma A, Ahmad R, Krishnan M, Washington MK, et al. Claudin-1 regulates intestinal epithelial homeostasis through the modulation of Notch-signalling. *Gut*. (2014) 63:622-34. doi: 10.1136/gutjnl-2012-304241

41. Pope JL, Ahmad R, Bhat AA, Washington MK, Singh AB, Dhawan P. Claudin-1 overexpression in intestinal epithelial cells enhances susceptibility to adenamatous polyposis coli-mediated colon tumorigenesis. *Mol Cancer*. (2014) 13:167. doi: 10.1186/1476-4598-13-167

42. Gong Y, Sunq A, Roth RA, Hou J. Inducible expression of claudin-1 in glomerular podocytes generates aberrant tight junctions and proteinuria through slit diaphragm destabilization. *J Am Soc Nephrol*. (2017) 28:106-17. doi: 10.1681/ASN.2015121324

43. Ahmad R, Chaturvedi R, Olivares-Villagómez D, Habib T, Asim M, Shivesh P, et al. Targeted colonic claudin-2 expression renders resistance to epithelial injury, induces immune suppression, and protects from colitis. *Mucosal Immunol*. (2014) 7:1340-53. doi: 10.1038/mi.2014.21

44. Turksen K, Troy TC. Permeability barrier dysfunction in transgenic mice overexpressing claudin 6. *Development*. (2002) 129:1775-84.
